# IL-33/ST2 signalling in pediatric food allergy and eosinophilic gastrointestinal disorders: a systematic review of human and preclinical evidence

**DOI:** 10.3389/fimmu.2026.1961426

**Published:** 2026-10-12

**Authors:** Monica Grama, Maria Oana Săsăran, Ana Stan Muntean, Delia Coman, Alina Ivasko, Cristina Oana Mărginean

**Affiliations:** 1 Doctoral School, “George Emil Palade” University of Medicine, Pharmacy, Science and Technology of Târgu Mures, Targu Mures, Romania; 2 Pediatric Clinic I, County Emergency Hospital of Targu Mures, Targu Mures, Romania; 3 Department of Pediatrics 3, “George Emil Palade” University of Medicine, Pharmacy, Sciences and Technology of Târgu Mures, Târgu Mures, Romania; 4 Department of Internal Medicine, County Emergency Hospital of Targu Mures, Targu Mures, Romania; 5 Department of Pediatrics 1, “George Emil Palade” University of Medicine, Pharmacy, Sciences and Technology of Târgu Mures, Târgu Mures, Romania

**Keywords:** alarmin triad, food allergies, interleukin 25, interleukin 33, pediatric patients, TSLP

## Abstract

**Background:**

Interleukin-33 (IL-33) is an epithelial-derived member of the IL-1 cytokine family that functions as an alarmin and signals through the ST2 receptor, upstream of type 2 inflammation. Together with thymic stromal lymphopoietin (TSLP) and IL-25, it constitutes an epithelial alarmin triad that orchestrates barrier-related immune responses. Despite substantial mechanistic data, the specific role of IL-33 as a biomarker and therapeutic target in pediatric food-related allergic disease remains insufficiently defined.

**Objective:**

This systematic review aims to synthesize current evidence on serum and tissue IL-33 in pediatric IgE-mediated food allergy and eosinophilic gastrointestinal disorders (EGIDs), to delineate its association with disease severity, clinical phenotype and oral immunotherapy (OIT) outcomes, and to critically appraise the IL-33/ST2 axis as a candidate biomarker and therapeutic target within the broader epithelial alarmin network.

**Methods:**

A systematic search of PubMed/MEDLINE, Web of Science, Scopus and the Cochrane Library (2010–2026) identified pediatric clinical studies quantifying IL-33 or IL-33/ST2 in food allergy or EGIDs, alongside murine and *in vitro* studies directly interrogating IL-33-dependent mechanisms in food allergy models. Eligible studies were screened and extracted according to predefined PICO criteria.

**Results:**

Fourteen studies fulfilled the inclusion criteria (four pediatric clinical cohorts, ten direct preclinical murine or *in vitro* studies). Across murine models, keratinocyte-derived IL-33 consistently activated a skin-to-gut axis driving intestinal mast-cell expansion and IgE-independent food anaphylaxis, which was abrogated by genetic or pharmacologic ST2 blockade. In a small case-control study of nine children with eosinophilic gastroenteritis, serum IL-33 and TSLP were increased during active disease and declined after dietary elimination. In pediatric eosinophilic esophagitis, IL-33 was increased in tissue but not consistently in serum. Conversely, serum IL-33 demonstrated limited discriminatory value in IgE-mediated food allergy.

**Conclusions:**

IL-33 emerges as a context-dependent yet non-redundant component of the epithelial alarmin circuitry in pediatric food-related allergic disease, as candidate biomarker of epithelial inflammation in pediatric EGIDs. However, current clinical evidence is limited and requires validation in larger prospective cohorts.

## 1 Introduction

### 1.1 Background on IL-33 biology

Interleukin 33 (IL-33) is a unique cytokine belonging to the IL-1 superfamily, described as a “tissue-derived nuclear cytokine”, which is abundantly expressed in endothelial and epithelial cells (1). Due to its predominant presence in structural barrier cells, such as keratinocytes and endothelial cells, it serves as the primary host-environment interface, and represents the first cytokine response under stress, and the primary sensor of mucosal integrity. A defining characteristic of IL-33 is its alarmin function. Working as a danger-associated molecular pattern (DAMP) (1–3), it is secreted upon necrosis, tissue damage, or, recently proven, environmental stressors, such as cold exposure in the case of mechanical skin injuries (1, 2). Released IL-33 binds to ST2L, the transmembrane isoform of IL1RL1. The IL-33/ST2L complex recruits IL-1 receptor accessory protein and activates MyD88-dependent signaling pathways, including IRAK and TRAF6. In contrast, soluble ST2 (sST2) acts as a decoy receptor that binds extracellular IL-33 and limits its signaling activity (4, 5). Ultimately, these processes will drive the production of type 2-associated cytokines, immune cell recruitment, and massive mast cell degranulation (6, 7).

### 1.2 IL-33 in food allergy

Food allergy is a typical type 2 immune disorder in which re-exposure to the allergen and epithelial injury activate the alarmin cascade. This reaction amplifies the IgE-mediated effector responses through cytokines such as IL-33, IL-25, and thymic stromal lymphopoietin (TSLP). Recent studies emphasize the important role IL-33 plays as an upstream mediator, particularly at barrier sites such as the skin and gut (8–10). In recent murine models, cutaneous exposure to food allergens on experimentally tape-stripped skin, designed to mimic atopic dermatitis-like lesions, led to robust IL-33 expression, as noted upon keratinocyte analysis (11). This mechanism afterwards triggers systemic circulation of IL-33, which promotes intestinal mast cell expansion, thereby predisposing the gut to severe food anaphylaxis and recurrent food allergy symptoms, upon subsequent oral ingestion (12–15). The same murine models have demonstrated attenuated oral anaphylaxis after oral food challenges (OFC), if genetic deletion of the ST2 receptor or antibody-mediated ST2 blockade occurred. In these models, serum levels of allergen specific IgE antibodies and type 2 cells remain intact, indicating that IL-33 detains an indispensable role in the sensitization phase, as well as a critical role in the effector phase of gut allergic/anaphylactic reactions (6, 11, 16).

### 1.3 Knowledge gaps

Despite abundant experimental data implicating IL-33 in the type 2 inflammation cascade, its exact role in human food allergies or hypersensitivities, particularly in child populations, remains unclearly defined. Existing clinical studies are small and heterogeneous in design, often combining pediatric and adult populations, thus making it difficult to establish direct correlations between cytokine levels, possible phenotypes, and serum pediatric ranges for the cytokine. Consequently, it remains unclear whether serum IL-33 can reliably predict the severity of gastrointestinal (GI) food allergies (FA), or if it can only indicate the acute onset of alarmin release and systemic inflammation that occurs afterwards (11, 17, 18). Most of the current literature clinical studies come from murine models. At the same time, human data is comprised of mostly one time serum levels of IL-33 or tissular IL-33 depicted from esophageal biopsies in eosinophilic esophagitis (EoE) patients (8). The resulting lack of well-defined human studies analyzing serum TSLP, IL-33, and IL-25, in comparison with pediatric cohorts limits the ability to define a well-established role of IL-33 and the associated phenotypes (8, 19, 20). Data on IL-33 from small studies and observational trials found that subjects with higher or imbalanced circulating IL-33/soluble-ST2 ratios presented more severe systemic symptoms or polysensitization (21). Mucosal biopsies consistently demonstrate the local elevation of IL-33 m RNA in the gastrointestinal and oral mucosa of allergic patients, but correlation with higher IL—33 peripheral blood levels has been inconsistently proven. For example, studies conducted on pediatric patients with eosinophilic esophagitis have shown a discordance between high local IL-33 levels and frequently normal cytokine serum levels (22). Although IL-33 and other related epithelial alarmins have not been sufficiently studied for their potential role as biomarkers in food allergies, biologic therapies directly targeting these pathways are already approved for clinical use in other type 2 inflammatory diseases such as atopic dermatitis, asthma, chronic rhinosinusitis with nasal polyposis and eosinophilic esophagitis (23, 24). Anti-TSLP antibodies such as Tezepelumab, antibodies targeting IL-33 (Itepekimab) and anti-ST2 antibodies (Astegolimab) have so far demonstrated reductions in asthma exacerbations frequency and in serum inflammatory markers levels, with acceptable safety profiles (25, 26). However, Tezepelumab is currently used in moderate and severe asthma treatment, and has only been validated as a therapeutic agent in children above 12 years of age (26). Itepekimab and Astegolimab are currently undergoing clinical trials for therapeutic validation in adults with chronic obstructive pulmonary disease (COPD) ( (25, 27), and have not yet been researched on pediatric populations. Hence, none of these monoclonal antibodies have been yet validated on pediatric cohorts, these findings providing only a preclinical therapeutic hypothesis. In the pediatric food-allergy setting, the lack of systematic clinical evaluation of alarmin-targeted biologics remains a major knowledge gap, leaving uncertainties regarding which children should receive targeted biologic treatment, how to best monitor their response to treatment and what long-term changes these therapies induce (28).

### 1.4 Rationale and objectives

Preclinical work in mice demonstrates that the epithelial alarmin triad (TSLP, IL-25, IL-33) is both necessary and sufficient to drive food-allergic responses (29, 30). In a murine medium-chain triglyceride (MCT)/egg-white model, prophylactic administration of monoclonal antibodies targeted against IL-25, the IL-33 receptor, or TSLP, before oral sensitization, markedly reduced the development of IgE-mediated food allergy, indicating that each cytokine contributes to the initiation of disease (29). When the same antibodies were given after food allergy was established, blockade of any single alarmin was insufficient to reverse disease, whereas combined targeting of all three pro-Th2 cytokines led to loss of clinical reactivity and induction of oral tolerance. These data support a model in which TSLP, IL-25 and IL-33 act in a partially redundant, cooperative fashion to maintain established food allergy and at the same time show that simultaneous inhibition of the triad can both prevent and suppress disease *in vivo* (29, 31). Complementary studies in TSLP-driven atopic-march models have shown that therapeutic blockade of ST2 after percutaneous sensitization reduces intestinal mast-cell expansion and diarrheal responses after oral challenge, highlighting that IL-33 represents a key effector-phase driver of gastrointestinal allergy (8). Together, these findings deliver proof that targeting IL-33 and its companion alarmins is a rational therapeutic strategy in food allergy, even though the interventions performed so far consisted of stand-alone antibody treatments after oral antigen exposure rather than as adjuncts to formal oral immunotherapy.

In contrast, clinical oral immunotherapy protocols for IgE-mediated food allergy in children predominantly rely on allergen administration alone or in combination with anti-IgE monoclonal antibodies, with alarmin targeted biologics being not yet integrated into routine practice. Pediatric randomized and observational studies show that oral immunotherapy response (OIT) to cow’s milk, egg and peanut can increase the threshold of reactivity, but they also report frequent allergic reactions during up dosing and maintenance. Anti-IgE therapy with omalizumab has been used as an adjunct to OIT to reduce the number and severity of reactions and to accelerate the build-up phase, yet it does not consistently increase the ultimate rate of sustained desensitization. Contemporary reviews of biologic therapy in food allergy focus largely on Omalizumab, Ligelizumab and Dupilumab, and do not report completed trials using antibodies directed against IL-33, TSLP or IL-25 in combination with OIT. Thus, despite strong mechanistic justification and encouraging preclinical efficacy, alarmin-targeted biologics have not yet been integrated into OIT protocols in humans (32–34).

Considering this background, the primary objective of this review is to synthesize current evidence on serum and tissue IL-33 in pediatric food-related allergic disease, with a focus on IgE-mediated food allergy and eosinophilic gastrointestinal disorders. We aim to describe how IL-33 levels relate to disease severity, clinical phenotype and atopic multimorbidity, and, where available, to compare IL-33 with TSLP and IL-25 within the epithelial alarmin triad. Secondary objectives were to synthesize preclinical evidence on the role of IL-33/ST2 signalling in sensitisation and effector responses and to appraise the translational potential of this pathway as a candidate biomarker and therapeutic target in pediatric food-related allergic disease.

## 2 Methods

### 2.1 Eligibility criteria (PICO framework): population-intervention-comparator-outcome

This systematic review was conducted in accordance with the Preferred Reporting Items for Systematic Reviews and Meta-Analyses (PRISMA) 2020 statement (35).

The review question was structured using the PICO framework. For the Population, two separate review questions were defined: a primary clinical question addressing pediatric human evidence and a secondary mechanistic question addressing preclinical evidence. The corresponding eligibility frameworks are presented in **Table 1**. The Intervention/Exposure was measurement or experimental modulation of IL-33 or the IL-33/ST2 axis, with TSLP and IL-25 considered where co-reported. The Comparator included healthy or non-allergic controls, other atopic conditions, distinct phenotypic or severity groups, or pre- versus post-treatment states. The Outcomes of interest were quantitative IL-33 levels and related IL-33/ST2 measures, and their associations with diagnosis, disease activity, severity, atopic multimorbidity, or responses to oral immunotherapy and biologic therapies. Human studies were eligible only if they enrolled children and adolescents aged 0–18 years or reported extractable pediatric subgroup data. Studies enrolling mixed pediatric and adult populations were excluded when pediatric-only data could not be extracted. Preclinical murine studies were eligible for the mechanistic synthesis only when they directly interrogated the IL-33/ST2 axis in a food-allergy or food-related eosinophilic gastrointestinal disease model. Direct mechanistic evidence was defined as genetic deletion or modification of IL-33 or ST2/IL1RL1, pharmacological blockade or neutralization of IL-33/ST2 signaling, recombinant IL-33 administration, or a prespecified mechanistic assessment focused on the IL-33/ST2 pathway. Studies reporting IL-33 only as one component of a broader inflammatory panel, without direct manipulation or focused mechanistic evaluation of the IL-33/ST2 axis, were excluded.

**Table 1:** PICO element description for pediatric and preclinical studies.

PICO element	Pediatric clinical question	Preclinical mechanistic question
*Objective*	To assess the association of IL-33/ST2-related measures with disease phenotype, activity, severity, and biomarker performance in pediatric food-related allergic disease	To examine the mechanisms through which IL-33/ST2 signalling contributes to sensitisation, effector responses, epithelial-barrier dysfunction, and oral tolerance in experimental food-allergy and food-related eosinophilic gastrointestinal disease models
*Population/model*	Children and adolescents aged 0–18 years with IgE-mediated food allergy or food-related eosinophilic gastrointestinal disorders, including eosinophilic oesophagitis and eosinophilic gastroenteritis	Animal, *in-vitro*, and ex-vivo models relevant to food allergy or food-related eosinophilic gastrointestinal inflammation
*Exposure/intervention*	Measurement of IL-33, ST2L, sST2, or IL-33/sST2-related measures in serum, plasma, saliva, tissue, or cells; co-measurement of TSLP and/or IL-25 when available	Genetic, pharmacological, recombinant-cytokine, cellular, or other experimental manipulation of IL-33, ST2/IL1RL1, or an IL-33-related pathway
*Comparator*	Healthy or non-allergic controls; atopic controls; active versus remission disease; phenotypic/severity groups; pre- versus post-treatment states	Wild-type versus knockout models; vehicle or isotype controls; untreated versus treated conditions; sensitised versus non-sensitised models; relevant *in-vitro* control conditions
*Outcomes*	IL-33/ST2/sST2 levels and their association with diagnosis, disease phenotype, activity, severity, biomarker performance, or treatment response	Anaphylaxis, diarrhoea, mast-cell, ILC2, and eosinophil responses; IgE and cytokine profiles; epithelial-barrier function; intestinal permeability; and oral-tolerance outcomes
*Eligible study designs*	Observational human studies and clinical trials with pediatric-only data or extractable pediatric subgroup data	Animal intervention studies and mechanistic *in-vitro*/ex-vivo experiments relevant to the prespecified conditions
*Key exclusion criteria*	Adults-only populations; mixed pediatric/adult populations without extractable pediatric data; studies lacking relevant IL-33/ST2-related outcomes	Studies that report IL-33 solely as an incidental marker in a broad inflammatory panel without relevant mechanistic analysis of the IL-33/ST2 pathway; models unrelated to food allergy or food-related EGID

IgE, immunoglobulin E; IL-25, interleukin-25; IL-33, interleukin-33; OIT, oral immunotherapy; PICO, Population, Intervention, Comparator, Outcomes; ST2, suppression of tumorigenicity 2 (IL-33 receptor); TSLP, thymic stromal lymphopoietin.

The overall study selection pathway is depicted in a PRISMA flowchart, depicted in the **Supplementary Materials**, **Figure 1**, whereas **Table 1** enumerates a detailed summary of the main methodological and clinical characteristics, based on the PICO elements of the included studies.

**Figure 1:**
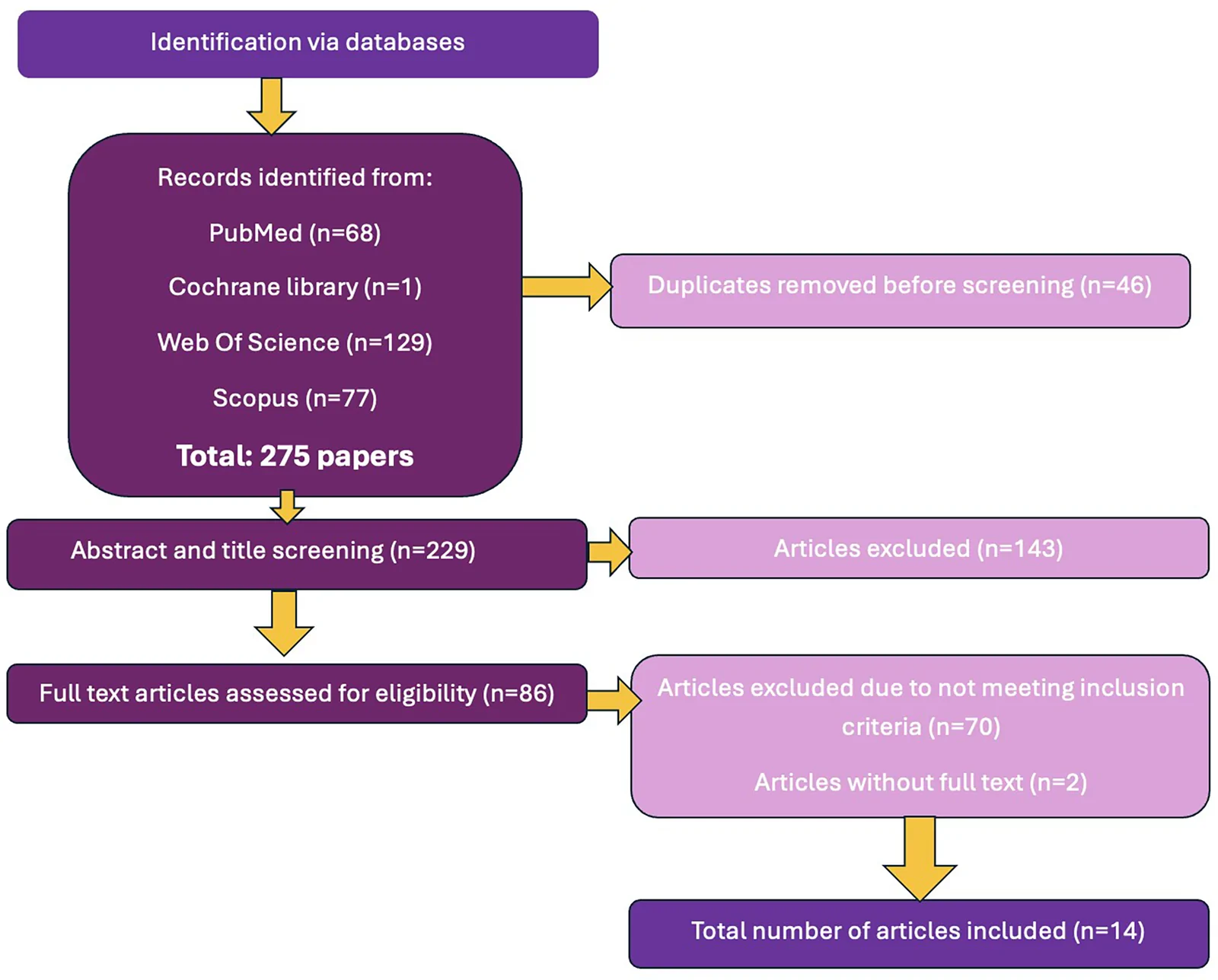
Prisma flowchart representing the selection process of eligible studies. n, number of records.

### 2.2 Database search strategy and study selection process:

We performed a systematic literature search in 4 major databases: PubMed/MEDLINE, Web of Science, Scopus and Cochrane Library in order to identify studies evaluating IL-33 in food related allergic disease, in children aged 0–18 years and murine models. Searches covered all records from 01.01.2010 to 17.04.2026. The search strategy combined controlled vocabulary (e.g. MeSH/Emtree) and free-text terms for IL-33 and the epithelial alarmins, with terms for food allergy and eosinophilic gastrointestinal disease. A core PubMed strategy was: (“interleukin-33” OR “IL-33” OR “IL33” OR “ST2” OR “IL1RL1”) AND (“food allergy” OR “food anaphylaxis” OR “oral immunotherapy” OR “food hypersensitivity” OR “eosinophilic esophagitis” OR “eosinophilic gastroenteritis” OR “eosinophilic gastrointestinal”). Specific search strings were used in accordance to each database searched, while preserving the same conceptual structure. The search was limited to English-language only articles, with no other additional restrictions. Human pediatric studies were prioritized for the primary objective, while animal and *in vitro* murine studies were included if these directly addressed IL/ST2 mechanisms relevant to food allergy or food-related eosinophilic gastrointestinal disease. After the initial search, the title and abstract screening was managed using Rayyan’s web-based platform, which facilitated duplicate detection, blinded decisions and conflict resolution. Full-text PDFs for potentially eligible studies were then retrieved and assessed manually to determine final inclusion and to record exclusion reasons (36).

All records retrieved from PubMed/MEDLINE, Web of Science, Scopus, and the Cochrane Library were exported to Rayyan for duplicate detection and screening. Title and abstract screening and full-text eligibility assessment were conducted using Rayyan according to prespecified eligibility criteria. Rayyan was used to facilitate duplicate detection, blinded decisions, and the management of screening conflicts. Authors MG, MOS, ASM and COM proceeded with the examination of the title and abstract of each identified article, in order to rule out those reports which did not comply with the reviewer’s objectives. MG, MOS, DC, AI and COM afterwards evaluated the full-length text of each of the papers elected in order to check whether each of them complied to the inclusion criteria mentioned above. Discrepancies identified during this verification were resolved by discussion and consensus. Each individual record included in this review was subject to mutual agreement from all authors.

### 2.3 Data extraction methods

Data were extracted independently from each paper. For human studies, we recorded: study design, country and setting, population characteristics (age range, diagnostic criteria for food allergy or eosinophilic gastrointestinal disease, atopic comorbidities), sample size, type and timing of IL-33 measurement (serum, plasma, or tissue; assay platform; units), co-measurement of TSLP and/or IL-25 where available, comparator groups, clinical outcomes (presence and severity of reactions, eosinophil counts, endoscopic/histologic scores), and main quantitative findings on associations between IL-33 (and related alarmins) and disease activity or treatment response.

For preclinical mechanistic animal studies, extracted items included: species and strain, sensitization and challenge protocols, route and timing of allergen exposure, interventions targeting IL-33, TSLP or IL-25, quantification of allergic disease (clinical scores, anaphylaxis, mast-cell and eosinophil responses, cytokine profiles), and key conclusions relevant to IL-33 biology in food allergy.

### 2.4 Data synthesis and risk of bias assessment

Given the substantial clinical and methodological heterogeneity across included studies, no meta-analysis was performed. Findings were synthesized narratively, stratified by evidence type (pediatric, clinical vs. preclinical evidence) and by biological compartment (serum, tissue, saliva), where applicable. Statistical pooling was not considered appropriate, due to differences in study design, assay platforms, and outcome measures.

Risk of bias was assessed separately for human and preclinical studies, because of their different study designs and potential sources of bias. The four pediatric observational studies were evaluated using the Newcastle–Ottawa Scale (NOS) case-control principles (37). The NOS awards a maximum of nine stars across three domains: Selection (maximum four stars), Comparability (maximum two stars), and Exposure (maximum three stars). Because the included human studies were comparative biomarker studies, the NOS case-control items were applied to case-control and cross-sectional designs. Scores of 7–9, 4–6, and 0–3 stars were interpreted as low, moderate, and high risk of bias, respectively.

The ten preclinical murine studies were assessed using SYRCLE’s Risk of Bias tool (38). The domains assessed were sequence generation, baseline characteristics, allocation concealment, random housing, blinding of caregivers and investigators, random outcome assessment, blinding of outcome assessors, incomplete outcome data, selective outcome reporting, and other sources of bias. Each domain was judged as low, high, or unclear risk of bias. “Unclear” was assigned when reporting did not provide sufficient information to determine whether a methodological safeguard had been used. Risk-of-bias assessments were considered in the narrative synthesis but were not used as exclusion criteria after study selection.

## 3 Results

### 3.1 Study selection

A total of 14 studies met the final inclusion criteria for this systematic review, comprising of human pediatric clinical studies and mechanistic murine *in vitro* models. The inclusion criteria captured a vast range of food-related allergic pathologies, with IgE-mediated food allergies and non-IgE-mediated eosinophilic gastro-intestinal disorders (EGIDs), such as eosinophilic esophagitis (EoE) and eosinophilic gastroenteritis (EGE). We included four human cohort studies (n=4) with diverse methodological designs, and different sample sizes. The biological matrices and quantification methods of IL-33 varied profoundly between the studies, exhibiting a profound heterogeneity. This lack of methodological standardization limited our ability to establish generalized pediatric reference ranges or to draw cohesive conclusions regarding baseline cytokine levels. Due to the lack of standardized human studies, we decided to also include *in vitro* murine models in order to draw conclusions regarding underlying mechanistic pathways of the IL-33/ST2 axis. We included ten (n=10) studies that used predominantly BALB/c and C57BL/6 wild type strains, alongside different interventions and other specific receptor knock-out variations, for alarmin function isolation. The overall study selection pathway is depicted in **Figure 1**.

### 3.2 Pediatric clinical evidence

The characteristics, biological compartments, analytical methods, and principal findings of the four included pediatric clinical studies are summarized in **Table 2**.

**Table 2:** Human pediatric studies assessing IL-33 and related epithelial alarmins in food-related allergic disease.

Study ID	Study type	Population	Allergen/Disease	Alarmins measured	Biological sample	IL-33 levels	Key findings
Judd et al., 2016 (22)	Case-control	n=9 pediatric EoE + 5 controls; age 5.3 ± 1.4 vs 9.65 ± 1.7 yrs	Eosinophilic esophagitis (EoE)	IL-33, CCL26, IL-5, IL-13 (mRNA and protein)	Esophageal biopsies (mRNA qRT-PCR; IHC); serum (ELISA)	IL-33 mRNA signal elevated in EoE esophageal mucosa(p<0.05); serum IL-33 was not elevated;	IL-33, CCL26, IL-5, IL-13 all significantly elevated in EoE biopsies. Tissue (not serum) IL-33 associated with EoE.
Shoda et al., 2016 (39)	Case-control + microarray	n=9 infantile EGE, 7 healthy controls, 37 FA (symptom-free), 31 AD (active), 8 UC (active); pediatric	Eosinophilic gastroenteritis (EGE)	TSLP, IL-33 (serum, 36-cytokine Milliplex); TSLP and IL33 mRNA (sigmoid colon)	Serum (Milliplex); sigmoid colon biopsies	TSLP and IL-33 both specifically elevated in active EGE serum (p<0.0001 vs all control groups); strong correlation r=0.91, p<0.0001; both present a decrease from active to convalescent phase	TSLP: sensitivity 77.8%, specificity 97.6% for EGE. IL-33: sensitivity 77.8%, specificity 95.2%. Both TSLP and IL33 mRNA were elevated in sigmoid colon in EGE vs UC.
Doherty et al., 2015 (40)	Case-control	n=15 active EoE, 12 inactive EoE, 3 PPI-REE, 3 non-EoE controls; pediatric, ages 1–17 yrs	Eosinophilic esophagitis (EoE)	IL-33, TSLP (ILC2 activation in culture)	Esophageal biopsies (flow cytometry); lymphoid aggregate culture	IL-33 and TSLP together expand ILC2s from esophageal lymphoid aggregates in culture; both alarmins required for maximal ILC2 activation	ILC2s (enriched in active EoE. ILC2 numbers correlate with eosinophil count (r=0.7, p<0.0001). IL-33 and TSLP synergistically expand esophageal ILC2s.
Ho et al., 2021 (41)	Cross-sectional, multidimensional	n = 105 (56 peanut allergy, 49 healthy controls); median age 10 yrs	Peanut allergy	IL-33, TSLP, IL-4, IL-5, IL-13 (salivary)	Saliva	IL-33 elevated in peanut allergy saliva (not statistically significant alone); TSLP also elevated trend; IL-4 significantly elevated (p=0.04)	↓ oral Prevotella/*Veillonella* correlated with ↓ SCFA and ↑ IL-33/TSLP. ↑ Neisseria correlated with Th2 milieu. Oral microbiome –metabolite – immune axis identified.

AD, atopic dermatitis; CBMC, cord blood mononuclear cell; CD4+, cluster of differentiation 4-positive (T cell); ELISA, enzyme-linked immunosorbent assay; EGE, eosinophilic gastroenteritis; EoE, eosinophilic esophagitis; FA, food allergy; gw, gestational week; IHC, immunohistochemistry; IL-4/IL-5/IL-13, interleukin-4/5/13; IL-33, interleukin-33; ILC2, group 2 innate lymphoid cell; n, number of participants; NCT, National Clinical Trial identifier (ClinicalTrials.gov); OIT, oral immunotherapy; PPI-REE, proton pump inhibitor-responsive esophageal eosinophilia; qRT-PCR, quantitative reverse transcription polymerase chain reaction; RCT, randomized controlled trial; scRNAseq, single-cell RNA sequencing; SCFA, short-chain fatty acid; TGFβ, transforming growth factor beta; Th1/Th2/Th17, T helper 1/2/17 cell; Treg, regulatory T cell; TSLP, thymic stromal lymphopoietin; UC, ulcerative colitis.

#### 3.2.1 Eosinophilic gastrointestinal disorders

Across the four pediatric clinical studies, IL-33 and related epithelial alarmins displayed a distinct, phenotype-dependent profile. In infantile eosinophilic gastroenteritis, one small pediatric case-control study including nine affected children reported higher serum IL-33 and TSLP concentrations during active disease, than in healthy and disease-control groups. Both analytes declined during convalescence after dietary elimination, and tissue analysis showed higher colonic IL-33 and TSLP mRNA expression. The study reported sensitivity of 77.8% and specificity of 95.2% for IL-33 at an empirically selected cutoff. However, these estimates were derived from a single small case-control cohort and require cautious interpretation because of limited precision, potential spectrum bias, possible cutoff optimization, and the absence of external validation (39). Taking into consideration that EGE is non-IgE mediated, these findings indicate that for these patients, epithelial IL-33 secretion is a direct consequence of ongoing mucosal stress caused by food antigen exposure, its role as a potential disease activity biomarker being suggested by the decline observed after dietary elimination (39). In eosinophilic esophagitis, mucosal—but not systemic—IL-33 characterized active disease: esophageal biopsies showed increased IL-33 mRNA alongside CCL26, IL-5 and IL-13, while circulating IL-33 remained within normal range, and esophageal ILC2 frequencies closely mirrored tissue eosinophil burden (22, 40). Functionally, ex vivo cultures demonstrated that IL-33 and TSLP act together to expand esophageal ILC2s, with neither cytokine alone eliciting comparable responses, which aligns with a model of non-redundant alarmin cooperation in sustaining eosinophilic mucosal inflammation (40). ILC2s were significantly expanded in esophageal biopsies from children with active EoE and their frequency correlated strongly with tissue eosinophilia (P < 0.0001) (40). Esophageal biopsies in children with active EoE revealed upregulated IL-33 mRNA signaling. Notably, serum IL-33 was not elevated, indicating a localized mucosal signal (42).

#### 3.2.2 IgE-mediated food allergy

In IgE-mediated peanut allergy, salivary IL-33 showed only a non-significant trend toward elevation, whereas classical Th2 cytokines such as IL-4 were more robustly increased and alarmin levels were intertwined with shifts in the oral microbiome and short-chain fatty acids, suggesting a more nuanced, microenvironment-dependent involvement of IL-33 (41). The intestinal and oral microbiome exert bidirectional regulatory influence on mucosal IL-33 signaling, establishing a mechanistic link between microbial dysbiosis and alarmin-driven food allergy. In children with peanut allergy, the salivary microbiome was characterized by increased abundance of *Neisseria* spp., which positively correlated with oral IL-33, TSLP, IL-5, and IL-13 secretion (P < 0.05) and was negatively associated with short-chain fatty acid (SCFA) levels. Conversely, *Prevotella* and *Veillonella* spp. were found to be enriched in healthy controls, and were positively associated with salivary acetate, butyrate, and propionate and negatively correlated with IL-33 and TSLP. These findings suggest that SCFA-producing commensal bacteria actively suppress mucosal alarmin secretion (41). In contrast, the discriminative value of serum IL-33 — alongside a broader panel of 15 Th17-related cytokines — is substantially limited in young atopic children with IgE-mediated food allergy. In a cohort of 76 children aged 0–5 years with chronic atopic dermatitis, all tested cytokines, simultaneously measured by multiplex Bio-Plex assay, including IL-33, showed a non-significant trend toward elevation in the IgE-mediated FA group compared with atopic controls without food allergy. Only IL-1β reached statistical significance (P < 0.05), with an area under the curve (AUC) of 0.67 on receiver operating characteristic (ROC) analysis. The suppression of IL-33 and Th17-related cytokines in this population is mechanistically interpretable: IL-4, which is constitutively elevated in active atopic dermatitis, strongly inhibits both Th17 polarization and IL-23 production, creating a Th2-dominant milieu that may attenuate the measurable serum signature of alarmins such as IL-33.

#### 3.2.3 Assay and compartment heterogeneity

As summarized in **Table 2**, the included studies differed substantially in clinical phenotype, biological compartment, comparator selection, and analytical platform. In children, atopic dermatitis is highly prevalent and associated with higher serum IL-33 levels compared to healthy controls. Taken together, the pediatric clinical evidence suggests that IL-33 measures are phenotype and compartment-dependent. Small studies support an association between local or circulating IL-33-related measures and eosinophilic gastrointestinal inflammation, whereas systemic and salivary findings in IgE-mediated food allergy are inconsistent. These data are insufficient to establish IL-33 as a validated diagnostic, prognostic, or treatment-monitoring biomarker in any pediatric food-related allergic condition (17, 43, 44).

IL-33 is not an established clinical biomarker in pediatric food-related allergic disease. The four included pediatric clinical studies were heterogeneous in clinical phenotype, biological compartment, comparator selection, assay platform, and timing of sampling. Shoda et al. examined serum IL-33 and TSLP, together with colonic mRNA expression, in infants with active eosinophilic gastroenteritis and multiple disease-control groups (39). Judd et al. assessed IL-33 in esophageal biopsy tissue and serum in children with eosinophilic esophagitis (22), whereas Doherty et al. quantified esophageal ILC2s by flow cytometry and evaluated IL-33/TSLP responsiveness in ex vivo lymphoid-cell cultures (40). Ho et al. measured salivary cytokines, including IL-33, using multiplex assays in children with peanut allergy and healthy controls (41). These differences preclude direct comparison of absolute IL-33 values and may partly explain why tissue IL-33 expression was increased in eosinophilic gastrointestinal disease while circulating or salivary IL-33 showed limited discriminatory value in IgE-mediated food allergy. The available evidence is limited to a small number of heterogeneous studies, most of which are cross-sectional or consist of case-control investigations with small patient groups.

Taken together, current clinical studies suggest that IL-33 may be a candidate biomarker of epithelial inflammation in selected pediatric eosinophilic gastrointestinal phenotypes. However, biological compartment, timing of sampling, assay platform, coexisting atopic disease, and the soluble ST2 milieu may substantially influence measured IL-33 concentrations. The evidence is insufficient to establish IL-33 as a clinically useful standalone biomarker (15).

### 3.3 Preclinical mechanistic evidence

The design, interventions and main mechanistic findings of the ten included murine and *in vitro* studies are presented in **Table 3**. Experimental murine and *in vitro* models collectively delineate IL-33 as a central, yet context-dependent, amplifier of food-allergic effector responses. Epicutaneous sensitization paradigms consistently identify the damaged epidermis as a dominant source of IL-33: barrier disruption induces a several-fold increase in cutaneous IL-33 expression, with subsequent release into both local tissue and circulation (11, 14). In these models, the presence or absence of functional ST2 signaling on mast cells decisively shapes clinical outcomes: oral anaphylaxis is abolished when mast cells are absent and re-emerges only when they are reconstituted with ST2-competent cells, while genetic or antibody-mediated ST2 blockade attenuates anaphylaxis severity and reduces IgE-mediated mast-cell degranulation (11). Intestinally, IL-33 operates through more than one effector route. One pathway involves joint activation of ILC2s by skin-derived IL-33 and tuft-cell–derived IL-25, driving IL-4/IL-13 production, mast-cell expansion and increased gut permeability, thereby lowering the threshold for food-induced anaphylaxis after oral challenge (14). A second, complementary pathway centers on IL-9–producing mucosal mast cells (MMC9s), which respond to IL-33 with marked hyperplasia; strains rich in intestinal MMC9s develop high rates of diarrhea upon oral allergen challenge, whereas strains lacking MMC9s remain clinically silent (12, 13). This MMC9 axis is reinforced by IL-4/IL-5 and depends on MHC class II–mediated antigen presentation, highlighting a tight link between alarmin signaling, antigen presentation and effector mast-cell proliferation (13).

**Table 3:** Murine and in vitro models investigating IL-33; TSLP and IL-25 in the pathogenesis of food allergies.

Study ID	Mouse model/Strain	Allergen used	Sensitization protocol	Alarmins measured	IL-33 levels	Key findings
Galand et al., 2016 (11)	BALB/c WT, ST2−/−	OVA (percutaneous + oral); TNP-BSA (passive)	3 cycles tape stripping + OVA application; oral OVA challenge day 49	IL-33 (serum + skin mRNA), OVA-IgE, mMCP-1, IL-4, IL-13	A rise of IL-33 mRNA of 3.89-fold was found in tape-stripped skin. IL-33 was released locally and systemically. ST2 deficiency significantly reduced anaphylaxis severity. IL-33 enhances IgE-mediated MC degranulation *in vitro*.	Oral anaphylaxis abrogated in MC-deficient mice and easy restored by WT-BMMC but not ST2−/− BMMC reconstitution. IL-33 acts directly on MCs.
Chen et al., 2015 (12)	BALB/c, A/J, C3H/HeJ, C57BL/6 (comparative)	OVA (intragastric)	IP OVA/Alum; 6× intragastric OVA challenges	IL-33, IL-9, IL-4, IL-13, IgE, MCPt-1	IL-33 activates MMC9s to produce large amounts of IL-9 and IL-13; IL-33 drives MMC9 effector function	Identified IL-9-producing mucosal mast cells (MMC9s). BALB/c mice (they had the highest MMC9 frequency) and were most susceptible to FA; C57BL/6 (no MMC9s) found to be resistant. MMC9 induction requires T cells, IL-4.
Oishi et al., 2025 (13)	BALB/c WT	OVA	OVA intragastric 20 mg × 7 challenges; MC reconstitution with WT or Ciita−/− BMMCs	IL-33, IL-9, IL-4, IL-5, MHCII, mMCP-1	IL-33 activates MMC9s to produce IL-9; IL-33-activated iMMCs are the source of excess IL-9 driving MMC hyperplasia	MHCII + MMCs present food antigens and then activate CD4+ T cells. IL-4/IL-5 axis enhances IL-9 by IL-33-activated MMCs (MMC9s), resulting in excessive MMC proliferation. MMC hyperplasia was suppressed in MHCII-deficient mice.
Leyva-Castillo et al., 2019 (14)	BALB/c abd C57BL/6	OVA (percutaneous); TNP-BSA (passive)	Tape stripping and IP OVA/Alum; oral OVA challenge	IL-33 (skin keratinocyte), IL-25 (gut tuft cell), ILC2, IL-4, IL-13	Elevation of IL-33 released from skin keratinocytes on mechanical injury; essential for intestinal MC expansion via ILC2 axis	Skin IL-33 and gut tuft cells IL-25 lead to ILC2 activation of IL-4/IL-13 axis, intestinal MC expansion and rise in intestinal permeability ultimately leading to food anaphylaxis. Both alarmins co-required.
Brandt et al., 2023 (20)	BALB/cJ, TSLPR−/−, ST2−/−	OVA ± Aspergillus fumigatus (percutaneous)	3 weekly percutaneous patches; 7× oral OVA challenge	IL-33, TSLP, IgE, MC degranulation	ST2−/− mice: partial reduction in IgE, MC degranulation, diarrhea. IL-33 contributes but is not dominant driver.	TSLP is the dominant driver: TSLPR−/− mice had NO diarrhea, near-absent intestinal MC accumulation, milder AD. IL-33/ST2 pathway was secondary to TSLP in this model.
Judd et al., 2016 (22)	BALB/c and C57BL/6	Aspergillus fumigatus (intranasal)	A. fumigatus 3×/week × 3 weeks; OR rIL-33 1 μg/day IP × 7 days	IL-33, ILC2, Th2, Treg, IL-13, eosinophils	IL-33 transiently induced in A. fumigatus EoE model	IL-33 drives EoE phenotype in a dependent manner alongside IL-13. IL-33-induced eosinophilia was abolished in IL-13−/− mice.
Li et al., 2020 (45)	BALB/c	OVA	OVA 10/20/50 μg IP/Alum days 0, 14; oral OVA 500 mg/L × 5 challenges; Bifidobacterium breve M-16V daily from day 0	IL-33, IL-4, IFN-γ, IgE, IgG, gut microbiota	FA mice: IL-33/ST2 enhanced signaling	FA mice show gut dysbiosis, and elevated IL-33/ST2, IgE/IgG, IL-4/IFN-γ ratios.
Liu et al., 2020 (46)	C57BL/6	OVA	Cold stress (4 °C × 1h/day × 7 days) + OVA gavage; challenge day 8.	IL-33 (intestinal IECs + serum), ACTH, cortisol, IL-4, IL-5, IL-13, IgE, mMCP-1	A rise of IL-33 via ACTH/cortisol axis and Il33 promoter demethylation under cold stress;	Cold stress activates ACTH/cortisol axis leading to Il33 promoter demethylation and a rise in IL-33. The effect is Th2 polarization and serum rise of IgE, cytokines.
Wang et al., 2024 (47)	BALB/c (female, 5–6 wks)	OVA vs BLG	IP OVA/Alum (days 0, 14); oral OVA 20 mg challenge (days 28–38)	IL-33, IL-4, IL-5, IL-13, IgE, mCPT-1	IL-33 signal decreased with oral OVA tolerance.	OVA oral tolerance led to lowering of: IgE, IL-33 via GSDMC pathway.
Rekima et al., 2020 (48)	BALB/c (pregnant/lactating dams + pups)	HDM (maternal) + OVA (pups)	Mothers intranasally exposed to HDM during lactation; pups breastfed; oral OVA tolerance assessed	IL-33 (gut mucosa), ILC2, FoxP3+/Tregs	A rise of IL-33 in gut mucosa of pups nursed by HDM-exposed mothers; HDM proteases in breast milk triggered IL-33 release	HDM proteases in breast milk lead to a rise in gut IL-33 and ILC2 activation. The result was Th2 priming inhibiting Treg/FoxP3 preventing the development of OVA oral tolerance. The result is FA susceptibility.

ACTH, adrenocorticotropic hormone; BALB/c, C57BL/6, A/J, C3H/HeJ, C3H/HeN, inbred mouse strains; BLG, beta-lactoglobulin; BMMC, bone marrow-derived mast cell; Ciita, class II major histocompatibility complex transactivator; CMA, cow’s milk allergy; FA, food allergy; FoxP3, forkhead box P3; GSDMC, gasdermin C; HDM, house dust mite; IECs, intestinal epithelial cells; IFN-γ, interferon gamma; IgE/IgG, immunoglobulin E/G; IL-4/IL-5/IL-9/IL-13, interleukin-4/5/9/13; IL-25, interleukin-25; IL-33, interleukin-33; ILC2, group 2 innate lymphoid cell; IP, intraperitoneal; MC, mast cell; MHCII, major histocompatibility complex class II; mMCP-1/mCPT-1, mouse mast cell protease-1; MMC9, interleukin-9-producing mucosal mast cell; mTOR, mechanistic target of rapamycin; OVA, ovalbumin; rIL-33, recombinant interleukin-33; ST2, suppression of tumorigenicity 2; TNP-BSA, trinitrophenyl-bovine serum albumin; Treg, regulatory T cell; TSLPR, thymic stromal lymphopoietin receptor; WT, wild-type.

#### 3.3.1 Skin-to-gut axis and epicutaneous sensitization

Murine models of percutaneous sensitization identify the epidermis as a major source of IL-33. Repeated tape-stripping or scratching-induced robust IL-33 expression in keratinocytes and later release, followed by a measurable rise in circulating IL-33 levels, which in turn drive the expansion of intestinal type 2 innate lymphoid cells (ILC2) and mast cells, thus lowering the oral threshold for oral tolerance after percutaneous ovalbumin (OVA) exposure (11, 14). Keratinocyte-specific IL-33 deletion (K14-Cre^Tg/0 Il33^flox/flox mice) abolished the serum IL-33 rise, intestinal ILC2 expansion, and subsequent food-induced anaphylaxis after percutaneous sensitization, directly attributing the effector consequence to skin-derived, rather than systemically circulating, IL-33 (11, 14).

Within the cutaneous context, TSLP and IL-33 roles appear to be stage dependent. Brandt et al. showed that in murine models, TSLP is essential for Th2 priming in systemic peanut allergies (20). Genetic and pharmacologic disruption of TSLP signaling reduced skin dendritic cell (DC) activation, peanut specific Th2 responses and serum IgE and IgG1 levels, and almost abolished clinical manifestations after oral peanut challenges. The same study proves that, in contrast, IL-33 or ST2 blocking during the same protocol, marks only modest improvement in clinical presentation (20). In line with these findings, OVA models with mechanical skin injury showed that IL-33 is a crucial element for amplifying mast-cell activation and promotion of anaphylaxis, after IgE sensitization (11, 14). Thus, while TSLP is the primary starter for the Th2 allergic pathway, IL-33 acts more prominently during the effector phase of the allergic cascade, and, this TSLP-dominant hierarchy appears context-dependent: in models of heavy mechanical barrier disruption (tape-stripping), keratinocyte-derived IL-33 becomes indispensable and acts together with TSLP, as demonstrated by the complete abrogation of intestinal mast cell expansion in keratinocyte-specific IL-33 knockout mice.

For further emphasis, a murine model was obtained, with exogenous administration of IL-33. This intervention was sufficient to induce core EoE features, such as transmural eosinophilia. Notably, IL-33 upregulation led to suppressed mucosal Foxp3, IL-10 and TGF-beta expression, suggesting that, beyond driving eosinophilic inflammation, it caused disturbances in local regulatory mechanisms.

#### 3.3.2 Mast-cell and ILC2-dependent pathways

The skin-gut model proved that IL-33 acted upstream of ILC2-dependent mast-cell expansion, by inducing the systemic release of keratinocyte-derived IL-33, in a synergistical manner with tuft-cell-derived IL-25, in order to activate intestinal ILC2 pathways and to further stimulate the production of IL-4, IL-13 (14). This enhanced intestinal permeability and anaphylaxis after oral allergen challenge, places IL-33 within a cytokine-mast-cell circuit, rather than as an effector of an isolated cytokine signal (11, 14).

Across multiple murine studies, IL-33 emerges as an amplifier of the allergic response, as proven by the lack of anaphylaxis responses upon oral challenges, found in ST2 deficient mice that were subjected to previous percutaneous sensitization. These clinical findings were backed by serum testing, which revealed that IL-33 potentiated antigen-driven IgE degranulation through ST2, and therefore induced synergistic IL-5 and IL-13 upregulation upon FcϵRI co-stimulation. These findings show that ST2 on mast-cells actually represents the receptor through which IL-33 lowers the threshold for food-induced anaphylaxis, after IgE sensitization (8, 14).

Beyond local mast cell activation, keratinocyte-derived IL-33 operates systemically through a skin-to-gut axis. Mechanical skin injury releases IL-33 into the circulation, which synergizes with intestinal tuft cell–derived IL-25, to expand intestinal ILC2s; ILC2-derived IL-4 then drives jejunal mast cell expansion, increasing intestinal permeability and anaphylaxis severity, independently of IgE titer (8, 14).

After reaching the intestinal mast cell compartment, IL-33 acts on a functionally distinct subset, namely IL-9–producing mucosal mast cells (MMC9s), to drive local mast-cell expansion and diarrhea. In BALB/c mice, which harbor high intestinal MMC9 frequencies, oral allergen challenge resulted in diarrhea in 91.7% of animals, whereas C57BL/6 mice, which lack intestinal MMC9s, showed no food-allergic symptoms – a natural experiment in strain susceptibility that mirrors the clinical variability in food-allergy severity among patients with comparable IgE profiles. Whether the MMC9–IL-33 axis operates similarly in human food allergy remains uncertain. Taken together with the ILC2/IL-4 findings from the skin-to-gut model, these data support a model in which IL-33 operates through two parallel yet converging mast-cell–expanding pathways: an ILC2-dependent route driven by systemic keratinocyte-derived IL-33 and a local MMC9-dependent route driven by mucosal IL-33 (12, 13).

Beyond IgE-mediated food allergy, murine models demonstrate that the IL-33/ILC2 axis can sustain eosinophilic mucosal inflammation through an entirely antigen-independent mechanism. This antigen-independent ILC2 mechanism, established in murine models, may be relevant to the ILC2 expansion observed in pediatric EoE, although direct causal evidence in children is lacking (36). ILC2s were significantly expanded in esophageal biopsies from children with active EoE and their frequency correlated strongly with tissue eosinophilia (P < 0.0001) — a relationship that resembles the IL-33-driven ILC2/mast cell correlation seen in the intestinal models, but operating in a different tissue, and through eosinophils rather than having mast cells as the terminal effector (36). *In vitro*, IL-33 and TSLP synergistically expanded these esophageal ILC2s, with neither cytokine individually producing equivalent responses, paralleling the non-redundant IL-33/IL-25 synergy identified in the intestinal skin-to-gut model (40).

Importantly, not all models place IL-33 at the apex of the alarmin cascade: in certain epicutaneous sensitization systems TSLP emerges as the dominant upstream driver, with TSLPR deficiency nearly abrogating diarrhea and intestinal mast-cell accumulation, while ST2 deficiency produces only partial reductions in humoral and clinical readouts, indicating disease- and protocol-specific hierarchies among epithelial alarmins (20).

#### 3.3.3 Environmental and microbial modifiers

The intestinal and oral microbiome exert bidirectional regulatory influence on mucosal IL-33 signaling, establishing a mechanistic link between microbial dysbiosis and alarmin-driven food allergy. In children with peanut allergy, the salivary microbiome was characterized by increased abundance of *Neisseria* spp., which positively correlated with oral IL-33, TSLP, IL-5, and IL-13 secretion (P < 0.05) and was negatively associated with short-chain fatty acid (SCFA) levels. Conversely, *Prevotella* and *Veillonella* spp. were found to be enriched in healthy controls, and were positively associated with salivary acetate, butyrate, and propionate and negatively correlated with IL-33 and TSLP. These findings suggest that SCFA-producing commensal bacteria actively suppress mucosal alarmin secretion. This inverse relationship between protective microbiota-derived metabolites and IL-33 was reinforced by intervention data which showed that Bifidobacterium breve M-16V, while not reducing intestinal IL-33 expression itself, significantly suppressed ST2 receptor expression in OVA-sensitized mice (P < 0.05). Further on, downstream Th2 and IgE responses were reduced, and gut microbial diversity was restored. Hence, IL-33/ST2 axis could possibly be modulated at the receptor level through microbial intervention, even when the alarmin source remains active (41, 45).

The included studies also identify triggers of intestinal epithelial IL-33 release that go beyond allergen exposure and mechanical barrier damage. Cold stress activated the ACTH/cortisol axis, inducing IL-33 promoter hypomethylation via NFATc1 in intestinal epithelial cells; the resulting Th2 responses and clinical food allergy were comparable in severity to those of conventionally sensitized mice, and were completely abolished in intestinal-epithelial-cell-specific IL-33 knockout mice (46). This confirms that epithelial IL-33, when released under physiological cold stress is, by itself, sufficient to drive food allergy in the absence of direct allergenic sensitization. The oral tolerance studies add a further layer. Early antigen-specific OVA exposure prevented food allergy by suppressing gasdermin C-mediated pyroptosis in intestinal epithelial cells, which directly reduced IL-33 secretion. In contrast, bystander antigen exposure expanded regulatory T cells, but left GSDMC expression and IL-33 levels unchanged, and provided no clinical protection from food allergies (47). These findings suggest that allergen-specific regulation of pyroptotic IL-33 release is a distinct and potentially critical step towards establishing oral tolerance.

Environmental and microbial factors further modulate IL-33 biology. Cold stress elevates intestinal epithelial and serum IL-33 via endocrine and epigenetic mechanisms, skewing Th2 responses and enhancing IgE and mast-cell mediators (46), whereas transfer of protease-rich allergens in breast milk rapidly increases gut mucosal IL-33 and ILC2 activation in offspring, impairing regulatory T-cell function and oral tolerance (48). Conversely, dietary nondigestible oligosaccharides and specific probiotic strains, such as Bifidobacterium breve M-16V, attenuate IL-33/ST2 signaling, rebalance Th2/Th1 cytokine ratios and reduce IgE and clinical scores (45), and the acquisition of oral tolerance to ovalbumin has been linked to downregulation of IL-33 via gasdermin-dependent pathways (46).

A maternal murine model study by Rekima et al. addresses a fundamental mechanism, describing how early-life allergen exposure through breast milk can prime the neonatal gut by the Th2 pathway,. This *in vivo* experiment used lactating BALB/c mouse models, sensitized with intranasal house mite (Der p), that shed Der p through breast milk, in substantial amounts, comparable to natural OVA proteins in human milk. A statistically significant rapid increase in IL-33 was identified (10-fold surge in the intestinal epithelium), entirely driven by the protease activity of Der p, leading to tight junction disruption and compromising the intestinal barrier integrity (48). This murine finding suggests a potential early-life mechanism relevant to food allergy susceptibility, though direct clinical translation remains unconfirmed.

### 3.4 Risk of bias assessment results

Risk-of-bias assessments are summarized in **Tables 4**, **5**. Using the Newcastle–Ottawa Scale, Ho et al. received 7 of 9 stars, indicating low-to-moderate risk of bias (41), whereas Shoda et al., Judd et al., and Doherty et al. received 5, 4, and 4 stars, respectively, indicating moderate risk of bias (22, 39, 40). The principal limitations across the human studies were incomplete reporting of case representativeness and non-response, limited control for confounding, and small or uneven comparator groups.

**Table 4:** Assessment of human study quality using the human clinical studies RoB- Newcastle-Ottawa Scale (NOS) (Observational/Case-Control & Cross-Sectional) tool.

Study	Selection: case definition adequate	Representativeness of cases	Selection of controls	Definition of controls	Comparability: age	Comparability: other confounders	Exposure/outcome ascertainment	Same method for cases/controls	Non-response rate reported/adequate	Total/9	Overall RoB
Shoda et al., 2016 (39)	★	0	★	★	0	0	★	★	0	5/9	Moderate
Judd et al., 2016 (22)	★	0	0	★	0	0	★	★	0	4/9	Moderate
Doherty et al., 2015 (40)	★	0	0	★	0	0	★	★	0	4/9	Moderate
Ho et al., 2021 (41)	★	0	★	★	★	★	★	★	0	7/9	Low-to-moderate

RoB, risk of bias; NOS, Newcastle–Ottawa Scale; RoB, risk of bias. A star (★) indicates that the study met the corresponding Newcastle–Ottawa Scale criterion; 0 indicates that the criterion was not met or was insufficiently reported. The maximum score was 9 stars: 4 for Selection, 2 for Comparability, and 3 for Exposure. For this review, scores of 7–9, 4–6, and 0–3 were interpreted as low, moderate, and high risk of bias, respectively. NOS was applied to comparative observational studies using the case-control version; the findings should be interpreted cautiously for cross-sectional designs.

**Table 5:** Assessment of murine studies using SYRCLE’s risk of bias tool – 10 key domains.

Murine study	D1 (Sequence	D2 (Baseline)	D3 (Allocation	D4 (Housing	D5 (Interv. Blinding)	D6 (Random eval.)	D7 (Outcome blinding	D8 (Incomplete data)	D9 (Reporting	D10 (Other bias)	Overall risk	Methodological summary & Key justifications
*Leyva-Castillo* et al.*, 2019 (*14*)*	*Low*	*Low*	*Unclear*	*Unclear*	*High*	*Unclear*	*Low*	*Low*	*Low*	*Low*	High risk	*Random allocation explicitly reported; blinded histological quantification of mast cells (CAE and tryptase); sex and age matched (female, 6–8 weeks*, *backcrossed >6 generations). High risk in D5: authors explicitly state experiments were performed without operator blinding.*
*Wang* et al.*, 2024 (*47*)*	*Unclear*	*Low*	*Unclear*	*Unclear*	*Unclear*	*Unclear*	*Low*	*Low*	*Low*	*Low*	Some concerns	*Diarrhea and fecal scores assessed blindly by 2 independent investigators; standardized SPF housing, 1-week acclimation, and OVA-free AIN-93G purified diet. Exact sequence generation method and allocation concealment are not described.*
*Brandt* et al.*, 2023 (*20*)*	*Unclear*	*Low*	*Unclear*	*Unclear*	*Unclear*	*Unclear*	*Unclear*	*Low*	*Low*	*Low*	*Some concerns*	*Automated, objective physiological readouts (TEWL probe, standardized flow cytometry panels); genetically matched cohorts bred in-house under SPF conditions. Lacks explicit statement regarding blinded histological mast cell counting.*
*Oishi* et al.*, 2025 (*13*)*	*Unclear*	*Low*	*Unclear*	*Unclear*	*Unclear*	*Unclear*	*Unclear*	*Low*	*Low*	*Low*	*Some concerns*	*Robust mast cell reconstitution model in Kit(W-sh/W-sh) mice using WT vs. Ciita^-^/^-^ cells (>8 generations backcrossing); male mice aged 6–12 weeks; repeated objective rectal temperature tracking. Allocation concealment and observer blinding not detailed.*
*Chen* et al.*, 2015 (*12*)*	*Low*	*Low*	*Unclear*	*Unclear*	*Unclear*	*Unclear*	*Low*	*Low*	*Low*	*Low*	*Some concerns*	*Explicit random group assignment (“mice were assigned at random”); food allergy parameters and diarrhea scores assessed in a strictly blinded fashion; formal pre-study sample size power calculation performed.*
*Galand* et al.*, 2016 (*11*)*	*Unclear*	*Low*	*Unclear*	*Unclear*	*Unclear*	*Unclear*	*Unclear*	*Low*	*Low*	*Low*	*Some concerns*	*Continuous automated body temperature logging via subcutaneous transponders (DAS-6001/IPTT-300); well-characterized female BALB/c cohorts (>9 backcrossed generations); all experiments independently replicated. Randomization sequence and histologic blinding not explicitly reported.*
*Liu et al., 2020 (* 46 *)*	*Unclear*	*Low*	*Unclear*	*Unclear*	*Unclear*	*Unclear*	*Unclear*	*Low*	*Low*	*Low*	*Some concerns*	*Standardized chronic cold stress protocol (4 °C, 1 h/day, 7 days); conditional intestinal epithelial Il33 knockout mice utilized; objective readouts (FITC-dextran 40 kDa, ChIP assays). Allocation concealment and evaluator blinding not detailed.*
*Li* et al.*, 2020 (*45*)*	*Low*	*Low*	*Unclear*	*Unclear*	*Unclear*	*Unclear*	*Low*	*Low*	*Low*	*Low*	*Some concerns*	*Randomization explicitly reported; CAE-stained small intestinal sections evaluated by observers blinded to sample identity; prior dose-response validation (10, 20, 50 µg OVA) before probiotic intervention.*
*Judd* et al.*, 2016 (*22*)*	*Unclear*	*Low*	*Unclear*	*Unclear*	*Unclear*	*Unclear*	*Low*	*Low*	*Low*	*Low*	*Some concerns*	*All quantitative morphometry and histologic cell counting were explicitly performed by a blinded observer; age-matched cohorts (10–12 weeks, WT vs. IL-13^-^/^-^); cross-validated across 2 independent experimental models (Aspergillus vs. recombinant IL-33). Random cage distribution not specified.*
*Rekima* et al.*, 2020[13.pdf] (*48*)*	*Unclear*	*Low*	*Unclear*	*Unclear*	*Unclear*	*Unclear*	*Low*	*Low*	*Low*	*Low*	*Some concerns*	*Tissue immunofluorescence (ZO-1, IL-33, E-cadherin) was quantified on an independent platform by a blinded operator; diarrhea scoring independently confirmed by 2 examiners; cross-fostering at birth prevented in utero maternal confounders. Sequence generation method not reported.*

D1, sequence generation; D2, baseline characteristics; D3, allocation concealment; D4, random housing; D5, blinding of caregivers and investigators; D6, random outcome assessment; D7, blinding of outcome assessors; D8, incomplete outcome data; D9, selective outcome reporting; D10, other sources of bias. Risk-of-bias judgments were classified as low, high, or unclear risk. Unclear risk indicates insufficient methodological information for judgment. SYRCLE, Systematic Review Centre for Laboratory animal Experimentation. Each domain was rated as low, high, or unclear risk of bias. “Unclear” indicates insufficient reporting to permit judgment and does not necessarily indicate that a procedure was not performed. Overall risk was judged as high where at least one domain was at high risk; otherwise, studies with one or more unclear domains were judged as having some concerns.

Ho et al. had clearly defined peanut allergy and healthy comparator groups and considered relevant potential confounders, although cytokine analyses were available only for a subset of salivary samples (41). Shoda et al. used histologically defined eosinophilic gastroenteritis and several disease-control groups but did not report adjustment for age or other clinical confounders; furthermore, colonic biopsies were obtained after dietary elimination, creating a temporal difference from baseline serum assessment (39). Judd et al. and Doherty et al. had small comparator groups and limited between-group comparability. In particular, the age difference between EoE cases and controls in the study of Judd et al. and the small non-EoE and PPI-REE groups in the study of Doherty et al. limit the strength of their between-group comparisons (22, 40).

Across the ten preclinical studies, several SYRCLE domains were judged as unclear because methods for sequence generation, allocation concealment, random housing, random outcome assessment, and blinding of caregivers or investigators were often not reported. Unclear reporting should not be interpreted as evidence that these procedures were not performed. Most studies reported comparable baseline characteristics, complete outcome data, and transparent outcome reporting. Several also incorporated methodological features that strengthen confidence in specific findings, including blinded histological assessment, objective physiological outcomes such as temperature monitoring, genetically modified mouse models, mast-cell reconstitution experiments, and replication across complementary experimental systems.

Overall, all preclinical studies were judged to have some concerns, primarily because of incomplete reporting of randomization, allocation, housing, and blinding procedures. The preclinical evidence nevertheless provides mechanistic support for IL-33/ST2 signaling in experimental food allergy and eosinophilic inflammation. These findings were interpreted as mechanistic and hypothesis-generating, rather than as direct evidence for causal pathways or therapeutic efficacy in children.

### 3.5 Translational synthesis

Pediatric clinical evidence in this review is limited to four small, heterogeneous studies. In infantile eosinophilic gastroenteritis, a single case-control study of nine affected children reported elevated serum and colonic IL-33/TSLP that declined after dietary elimination, but these diagnostic estimates require external validation before clinical use (39). In eosinophilic esophagitis, local mucosal IL-33 upregulation was consistently observed, whereas circulating IL-33 remained normal, and ex vivo cultures confirmed that IL-33 and TSLP synergistically expand esophageal ILC2s (22, 40). In IgE-mediated peanut allergy, salivary and serum IL-33 signals were weak and inconsistent, becoming informative only when integrated with oral microbiome and Th17-cytokine data (41).

By contrast, the ten included preclinical studies provide causal mechanistic evidence for IL-33/ST2 signaling that has not been directly tested in children. Genetic deletion of keratinocyte-derived IL-33 abolished food-induced anaphylaxis after percutaneous sensitization in mice (11, 14), and ST2-deficient mice failed to develop oral anaphylaxis despite intact IgE sensitization (11). The IL-9-producing mucosal mast-cell (MMC9) pathway, the epicutaneous skin-to-gut axis, and the modulatory roles of cold stress, maternal allergen exposure, and gut microbiota were likewise established exclusively in murine models (12, 13, 46, 48).

These two evidence streams should not be treated as equivalent. The pediatric clinical data are compatible with a compartmentalized, tissue-restricted pattern of IL-33 expression, particularly in eosinophilic gastrointestinal disease, but they do not confirm the causal skin-to-gut, ILC2-dependent, or MMC9-dependent pathways demonstrated in animal models. Preclinical findings should therefore be interpreted as informing biological plausibility and generating testable hypotheses for pediatric food allergy, rather than as confirmed human disease mechanisms.

## 4 Discussions

### 4.1 Interpretation of pediatric clinical evidence

The biomarker signal reviewed here is also shaped by a layer of receptor-level regulation that is rarely accounted for in the included clinical studies. Because soluble ST2 (sST2) acts as a decoy receptor that sequesters free IL-33 before it can engage membrane-bound ST2 (15), circulating IL-33 concentrations alone may not accurately reflect the biologically active alarmin signal reaching target cells (21). This framework may help explain why higher circulating IL-33 concentrations were observed in the small infantile eosinophilic gastroenteritis case-control study (39), whereas salivary or circulating IL-33 findings were inconsistent in IgE-mediated food allergy (41). However, sST2 was not consistently measured, and this proposed explanation remains speculative, where a higher relative sST2 burden could mask an otherwise active local signal. Future studies reporting the IL-33/sST2 ratio, rather than IL-33 concentration in isolation, may therefore better capture the effective alarmin tone across pediatric phenotypes.

### 4.2 Interpretation of preclinical evidence

Within this network, the murine data reveal that IL-33 does not act as a uniform upstream trigger but occupies distinct positions depending on the sensitization route and tissue context. In models of heavy epicutaneous barrier disruption, keratinocyte-derived IL-33 is indispensable and acts synergistically with tuft-cell-derived IL-25 to drive ILC2-dependent mast-cell expansion in the gut (11, 14), whereas in other percutaneous sensitization protocols TSLP emerges as the dominant initiator of Th2 priming, with IL-33/ST2 signaling contributing only a secondary, partial effect (20). A second, parallel effector route operates independently of ILC2s altogether, centered on IL-9-producing mucosal mast cells (MMC9s): strains with high intestinal MMC9 frequency develop severe diarrheal responses upon oral challenge, while MMC9-deficient strains remain clinically silent despite comparable IgE titers (12, 13). This duality—an ILC2-dependent circuit and a parallel MMC9-dependent circuit—suggests that IL-33 does not act through a single, linear pathway but through at least two convergent, partially redundant effector arms, which may explain why single-agent alarmin blockade has shown incomplete efficacy in preclinical prevention and treatment studies (29, 31). These findings also carry broader relevance for the atopic march. The demonstration that mechanical skin injury alone—independent of any oral allergen exposure—is sufficient to expand the intestinal mast-cell pool and lower the threshold for food-induced anaphylaxis provides a plausible mechanistic bridge between early atopic dermatitis and subsequent food allergy (11, 14). Murine models have allowed for accurate manipulation of the epithelial cytokine expression and their receptor pathways, through genetic deletion, receptor knockouts, external stress or downstream up-signaling. In contrast, even though human studies are more constrained, smaller and methodologically limited, their results emerge as compatible evidence with selected aspects of preclinical literature, particularly the compartmentalized nature of IL-33 expression in eosinophilic gastrointestinal inflammation. Together, these findings identify biologically plausible pathways that underline the translational relevance of epithelial alarmins and help steer medical knowledge towards possible therapeutic targets for food allergies and eosinophilic gastrointestinal disorders. Human and preclinical findings are complementary but represent distinct levels of evidence. These mechanistic and clinical observations frame the specific findings discussed in the remainder of this section, beginning with their convergence with the existing alarmin literature.

### 4.3 Translational implications and research gaps

The human and preclinical evidence streams should not be interpreted as equivalent. Genetic and pharmacological experiments in animal models provide mechanistic evidence for a causal role of IL-33/ST2 signaling under specific experimental conditions, whereas the pediatric human literature consists of a small number of heterogeneous observational studies. Therefore, the animal data inform biological plausibility and hypothesis generation but cannot establish clinical biomarker utility, therapeutic efficacy, or causal pathways in children.

The mechanistic findings synthesized in this review are supported by the existing alarmin literature, which has increasingly converged on a model in which TSLP, IL-33, and IL-25 act as non-redundant, context-dependent epithelial danger signals that collectively orchestrate type 2 immunity at barrier surfaces. A landmark study by Noti et al. demonstrated that while all three alarmins are simultaneously required for the induction of experimental food allergy, each single alarmin is independently sufficient to maintain an established disease. These preclinical findings have translational relevance and generate testable therapeutic hypotheses. However, they do not establish that IL-33 blockade, alone or in combination with other alarmin-targeted approaches, is clinically effective in pediatric food allergy (29). This hypothesis was further elaborated by Cayrol and Girard in their comprehensive review of the alarmin family, which classified IL-33 as a dual-function nuclear cytokine that, unlike TSLP and IL-25, possesses both intranuclear transcriptional regulatory functions and extracellular alarmin properties, conferring unique biology, not shared by its co-alarmins (49, 50). Annunziato et al., in their dedicated review on alarmins in food allergy, further identified a critical gap in the human pediatric data. While murine mechanistic evidence for IL-33 is robust, prospective clinical studies in children with food allergy remain sparse (51). Evidence from other pediatric inflammatory settings similarly implicates epithelial injury and the release of IL-33, IL-25 and TSLP in promoting Th2 polarization and ILC2 expansion, although direct extrapolation to food allergy requires caution (52).

### 4.4 Future research directions

A critical step before establishing IL-33 as a useful, clinical and accurate biomarker is represented by the identification of standardized age specific reference ranges for serum values of IL-33 and ST2, mainly for healthy children, but also for those with different allergic phenotypes of diseases. The age-dependent variation for allergen sensitization and polysensitization calls for the need of further research, to identify age-stratified pediatric reference intervals and phenotype-specific interpretation of epithelial alarmins (53). Currently, studies exhibit heterogeneity in terms of assay platforms used and measurement methods applied, sample timing, and other practicality issues. Thus, cross-study comparisons are unreliable (18, 54–56). Future investigations must systematically incorporate IL-33/sST2 ratios, as the decoy receptor sST2 substantially modulates the effective biological signal of circulating IL-33. Alongside standardization, the prospective validation of IL-33 as an oral immunotherapy (OIT) biomarker represents a high priority. For instance, the exploratory findings from a mixed-age POISED mechanistic sub-study provided preliminary evidence that allergen-specific T cell IL-33 expression at week 24 of OIT may predict desensitization outcomes (57). Validating this finding in pediatric-specific, multi-center OIT trials across various food allergens and younger age groups—where the immune landscape differs substantially from older cohorts—could enable the early identification of non-responders and directly impact protocol personalization (16, 58–60).

Beyond diagnosis, advancing alarmin-targeted biologics as OIT adjuncts require urgent attention. Preclinical models demonstrate that the combined blockade of TSLP, IL-25, and IL-33 can both prevent and suppress established food allergy (61–64). Given the established precedent of omalizumab-facilitated OIT, initiating such trials is crucial, particularly for polysensitized children burdened by severe atopic multimorbidity. In parallel, exploring microbiome–alarmin interactions delivers a promising take for early prevention. Whether this approach can improve the safety, desensitization outcomes, or sustained unresponsiveness of OIT in children remains unknown and should be assessed in appropriately designed early-phase clinical trials.

The inverse relationship between SCFA-producing commensals and mucosal IL-33 and TSLP, combined with the ST2-suppressing capacity of Bifidobacterium breve M-16V, provides a strong mechanistic basis for microbiome-modulating interventions in at-risk infants (45, 65). Longitudinal birth cohort studies incorporating serial alarmin measurements from the neonatal period through early childhood are needed to define the predictive value of early IL-33 trajectories for subsequent allergic outcomes. Finally, developing non-invasive mucosal IL-33 monitoring techniques is fundamental for managing eosinophilic gastrointestinal disorders (EGIDs), where tissue IL-33 acts as the primary signal, but serum levels often fail to reflect mucosal disease activity. Whether IL-33-related immune signatures can predict response to oral immunotherapy in children remains unknown and should be examined in adequately powered pediatric-only cohorts. Validating stool-based mucosal protein measurements or salivary alarmin panels could substantially reduce the diagnostic burden on children who currently require serial, repeated endoscopies for disease monitoring (66). Prospective validation of non-invasive biomarker panels, including blood-, stool-, and saliva-based measures, could reduce the need for repeated endoscopies with biopsies in children with eosinophilic esophagitis; however, these approaches are not yet validated for routine clinical decision-making (67, 68).

### 4.5 Data limitations

Notably, this review has a few limitations that need to be acknowledged. Firstly, the included human pediatric studies are characterized by small sample sizes and heterogenous designs, thus limiting the ability to perform meta-analyses or estimates for quantitative serum levels for IL-33, across different allergic phenotypes. In addition, the human clinical studies varied in the timing of IL-33 dosing, had different sites of sampling (GI tract, CNS, salivary, serum, plasma), varied in assay platforms and detection thresholds. The predominance of murine mechanistical data transcripts some conclusions through extrapolation from murine models to human pathways, with an immune architecture that may differ. Language restrictions to only English written clinical trials could have also introduced language bias, with potential exclusion of relevant studies. Lastly, another limitation would be the lack of longitudinal pediatric cohorts, especially regarding studies that simultaneously assess all three alarmins, with follow-up reevaluation after treatment or clinical intervention. This hinders definitive conclusions regarding the relative, temporal contributions of TSLP, IL-25, and IL-33 at different stages of food allergy development in children.

## 5 Conclusions

This review identifies biologically coherent but clinically limited evidence regarding IL-33/ST2 signaling in pediatric food-related allergic disease. Small pediatric studies suggest associations between IL-33-related measures and eosinophilic gastrointestinal inflammation; however, the current evidence does not establish IL-33 as a validated biomarker. Preclinical studies provide mechanistic support but require cautious translation to children. The translation of these findings into clinical practice will require prospective, pediatric-specific cohort studies with standardized alarmin measurement and longitudinal follow-up — a research agenda that is both feasible and timely, given current interest in precision endotypes in pediatric allergic disease.

## Data Availability

The original contributions presented in the study are included in the article/**Supplementary Material**. Further inquiries can be directed to the corresponding author.
