# Supplementary material for: IL-33/ST2 signalling in pediatric food allergy and eosinophilic gastrointestinal disorders: a systematic review of human and preclinical evidence
Source: Front Immunol. 2026 Oct 12;17:1961426. doi: 10.3389/fimmu.2026.1961426 (PMC13696261; doi:10.3389/fimmu.2026.1961426)
Supplement: Supplementary file 1 [file Table1.docx]

**Supplementary table 1.** Studies excluded after full text assesment.

| **Title** | **Year** | **Journal** | **Volume** | **Issue** | **Pages** | **Authors** | **doi** | **Exclusion criteria** |
| --- | --- | --- | --- | --- | --- | --- | --- | --- |
| The impact of different challenge protocols on immune response in epicutaneously sensitized mice with food allergy | 2025 | FOOD & FUNCTION | 16 | 12 | 4772-4783 | Wang, JS and Wang, XD and Gao, JY and Tong, P and Wu, Y and Li, X and Chen, HB | 10.1039/d5fo01045d | no IL-33 analysis |
| IBD functions as a double-edged sword for food allergy in BALB/c mice model | 2024 | IMMUNOLOGY | 173 | 2 | 394-407 | Chen, BH and Wu, YH and Wu, H and Gao, JY and Meng, XY and Chen, HB | 10.1111/imm.13833 | no IL-33 analysis |
| Efficacy and safety of food allergy oral immunotherapy in adults | 2023 | ALLERGY | 78 | 3 | 803-811 | Epstein-Rigbi, N and Levy, MB and Nachshon, L and Koren, Y and Katz, Y and Goldberg, MR and Elizur, A | 10.1111/all.15537 | wrong population |
| Primary prevention of food allergy in 2021: Update and proposals of French-speaking pediatric allergists | 2022 | ARCHIVES DE PEDIATRIE | 29 | 2 | 81-89 | Sabouraud-Leclerc, D and Bradatan, E and Moraly, T and Payot, F and Larue, C and Chabbert, AB and Nemni, A and Pontcharraud, R and Juchet, A and Divaret-Chauveau, A and Morisset, M | 10.1016/j.arcped.2021.11.009 | Wrong publication type |
| Epicutaneous immunotherapy for treatment of peanut allergy: Follow-up from the Consortium for Food Allergy Research | 2021 | JOURNAL OF ALLERGY AND CLINICAL IMMUNOLOGY | 147 | 3 | 992-+ | Scurlock, AM and Burks, AW and Sicherer, SH and Leung, DYM and Kim, EH and Henning, AK and Dawson, P and Lindblad, RW and Berin, MC and Cho, CB and Davidson, WF and Plaut, M and Sampson, HA and Wood, RA and Jones, SM and Consortium Food Allergy Res CoFAR | 10.1016/j.jaci.2020.11.027 | No IL-33 analysis |
| Analysis of Serum Th2 Cytokines in Infants with Non-IgE Mediated Food Allergy Compared to Healthy Infants | 2022 | NUTRIENTS | 14 | 8 | - | Savino, F and Giuliani, F and Giraudi, S and Galliano, I and Montanari, P and Daprà, V and Bergallo, M | 10.3390/nu14081565     WE  - Science Citation Index Expanded (SCI-EXPANDED) | No IL-33 analysis |
| Food Allergy in Infants With Atopic Dermatitis: Limitations of Food-Specific IgE Measurements | 2015 | PEDIATRICS | 136 | 6 | E1530-E1538 | Spergel, JM and Boguniewicz, M and Schneider, L and Hanifin, JM and Paller, AS and Eichenfield, LF | 10.1542/peds.2015-1444     WE  - Science Citation Index Expanded (SCI-EXPANDED) | No IL-33 analysis |
| Reported symptoms of food hypersensitivity and sensitization to common foods in 4-year-old children | 2008 | ACTA PAEDIATRICA | 97 | 1 | 85-90 | Ostblom, E and Wickman, M and van Hage, M and Lilja, G | 10.1111/j.1651-2227.2007.00556.x     WE  - Science Citation Index Expanded (SCI-EXPANDED) | No IL-33 analysis |
| Enhanced IL-4 but normal interferon-gamma production in children with isolated IgE mediated food hypersensitivity | 1998 | PEDIATRIC ALLERGY AND IMMUNOLOGY | 9 | 2 | 68-72 | Campbell, DE and Hill, DJ and Kemp, AS | 10.1111/j.1399-3038.1998.tb00306.x     WE  - Science Citation Index Expanded (SCI-EXPANDED) | No IL-33 analysis |
| Altered T Helper 17 Responses in Children with Food Allergy | 2013 | INTERNATIONAL ARCHIVES OF ALLERGY AND IMMUNOLOGY | 162 | 4 | 318-322 | Bin Dhuban, K and d'Hennezel, E and Ben-Shoshan, M and McCusker, C and Clarke, A and Fiset, P and Mazer, B and Piccirillo, CA | 10.1159/000354028     WE  - Science Citation Index Expanded (SCI-EXPANDED) | No IL-33 analysis |
| Food Allergy and Anaphylaxis in Infants and Preschool-Age Children | 2014 | CLINICAL PEDIATRICS | 53 | 7 | 652-657 | Gaspar-Marques, J and Carreiro-Martins, P and Papoila, AL and Caires, I and Pedro, C and Araújo-Martins, J and Virella, D and Rosado-Pinto, J and Leiria-Pinto, P and Neuparth, N | 10.1177/0009922814527502     WE  - Science Citation Index Expanded (SCI-EXPANDED) | No IL-33 analysis |
| Allergic Reactions to Foods in Preschool-Aged Children in a Prospective Observational Food Allergy Study | 2012 | PEDIATRICS | 130 | 1 | E25-E32 | Fleischer, DM and Perry, TT and Atkins, D and Wood, RA and Burks, AW and Jones, SM and Henning, AK and Stablein, D and Sampson, HA and Sicherer, SH | 10.1542/peds.2011-1762     WE  - Science Citation Index Expanded (SCI-EXPANDED) | No IL-33 analysis |
| Predictors of childhood food allergy: significance and implications | 2011 | ASIAN PACIFIC JOURNAL OF ALLERGY AND IMMUNOLOGY | 29 | 4 | 313-317 | Al-Hammadi, S and Zoubeidi, T and Al-Maskari, F |  | No IL-33 analysis |
| Clinical Perspectives of Food Allergy in Infants and Young Children | 2011 | PEDIATRIC GASTROENTEROLOGY HEPATOLOGY & NUTRITION | 14 | 2 | 113-121 | Hwang, JB | 10.5223/kjpgn.2011.14.2.113     WE  - Emerging Sources Citation Index (ESCI) | No IL-33 analysis |
| TLR4 receptor and selected cytokines in development of food allergy in infants and small children | 2011 | PRZEGLAD GASTROENTEROLOGICZNY | 6 | 6 | 370-375 | Kamer, B and Kulig, K and Lewkowicz, P and Glowacka, E and Sasiak, A and Tchórzewski, H | 10.5114/pg.2011.25992     WE  - Science Citation Index Expanded (SCI-EXPANDED) | No IL-33 analysis |
| Diagnosis and therapy of food allergy in infants and children | 2003 | MONATSSCHRIFT KINDERHEILKUNDE | 151 |  | S39-S46 | Niggemann, B | 10.1007/s00112-003-0790-2     WE  - Science Citation Index Expanded (SCI-EXPANDED)     WE  - Conference Proceedings Citation Index - Science (CPCI-S) | No IL-33 analysis |
| Early oral immunotherapy in peanut-allergic preschool children is safe and highly effective | 2017 | JOURNAL OF ALLERGY AND CLINICAL IMMUNOLOGY | 139 | 1 | 173-+ | Vickery, BP and Berglund, JP and Burk, CM and Fine, JP and Kim, EH and Kim, JI and Keet, CA and Kulis, M and Orgel, KG and Guo, RS and Steele, PH and Virkud, YV and Ye, P and Wright, BL and Wood, RA and Burks, AW | 10.1016/j.jaci.2016.05.027     WE  - Science Citation Index Expanded (SCI-EXPANDED) | No IL-33 analysis |
| Community-Based Adverse Food Reactions and Anaphylaxis in Children with IgE-Mediated Food Allergy at Age 6 Years: A Population-Based Study | 2020 | JOURNAL OF ALLERGY AND CLINICAL IMMUNOLOGY-IN PRACTICE | 8 | 10 | 3515-3524 | Wang, YC and Peters, RL and Perrett, KP and McWilliam, VL and Tang, MMLK and Lowe, AJ and Ponsonby, AL and Dharmage, SC and Allen, KJ and Koplin, JJ and HealthNuts Investigators | 10.1016/j.jaip.2020.07.008     WE  - Science Citation Index Expanded (SCI-EXPANDED) | No IL-33 analysis |
| Efficacy and safety of oral immunotherapy with AR101 in European children with a peanut allergy (ARTEMIS): a multicentre, double-blind, randomised, placebo-controlled phase 3 trial | 2020 | LANCET CHILD & ADOLESCENT HEALTH | 4 | 10 | 728-739 | O'BHourihane, J and Beyer, K and Abbas, A and Fernández-Rivas, M and Turner, PJ and Blumchen, K and Nilsson, C and Ibáñez, MD and Deschildre, A and Muraro, A and Sharma, V and Erlewyn-Lajeunesse, M and Zubeldia, JM and De Blay, F and Sauvage, CD and Byrne, A and Chapman, J and Boralevi, F and DunnGalvin, A and O'Neill, C and Norval, D and Vereda, A and Ben Skeel and Adelman, DC and du Toit, G | 10.1016/S2352-4642(20)30234-0     WE  - Science Citation Index Expanded (SCI-EXPANDED) | No IL-33 analysis |
| Cord blood monocyte-derived inflammatory cytokines suppress IL-2 and induce nonclassic "TH2-type" immunity associated with development of food allergy | 2016 | SCIENCE TRANSLATIONAL MEDICINE | 8 | 321 | - | Zhang, YX and Collier, F and Naselli, G and Saffery, R and Tang, MLK and Allen, KJ and Ponsonby, AL and Harrison, LC and Vuillermin, P and BIS Investigator Grp | 10.1126/scitranslmed.aad4322     WE  - Science Citation Index Expanded (SCI-EXPANDED) | No IL-33 analysis |
| Allergen specificity of early peanut consumption and effect on development of allergic disease in the Learning Early About Peanut Allergy study cohort | 2018 | JOURNAL OF ALLERGY AND CLINICAL IMMUNOLOGY | 141 | 4 | 1343-1353 | du Toit, G and Sayre, PH and Roberts, G and Lawson, K and Sever, ML and Bahnson, HT and Fisher, HR and Feeney, M and Radulovic, S and Basting, M and Plaut, M and Lack, G and Immune Tolerance Network Learning | 10.1016/j.jaci.2017.09.034     WE  - Science Citation Index Expanded (SCI-EXPANDED) | No IL-33 analysis |
| A Randomized, Open-Label Trial of Hen's Egg Oral Immunotherapy: Efficacy and Humoral Immune Responses in 50 Children | 2021 | JOURNAL OF ALLERGY AND CLINICAL IMMUNOLOGY-IN PRACTICE | 9 | 5 | 1892-+ | Palosuo, K and Karisola, P and Savinko, T and Fyhrquist, N and Alenius, H and Mäkelä, MJ | 10.1016/j.jaip.2021.01.020 | No IL-33 analysis |
| Food-induced anaphylaxis in children up to 3-years-old - preliminary study | 2021 | ALLERGOLOGIA ET IMMUNOPATHOLOGIA | 49 | 3 | 56-64 | Gawryjolek, J and Krogulska, A | 10.15586/aei.v49i3.77     WE  - Science Citation Index Expanded (SCI-EXPANDED) | No IL-33 analysis |
| Egg Yolk Provides Th2 Adjuvant Stimuli and Promotes Sensitization to Egg White Allergens in BALB/c Mice | 2018 | MOLECULAR NUTRITION & FOOD RESEARCH | 62 | 13 | - | Pablos-Tanarro, A and Lozano-Ojalvo, D and Martínez-Blanco, M and Molina, E and López-Fandiño, R | 10.1002/mnfr.201800057     WE  - Science Citation Index Expanded (SCI-EXPANDED) | No IL-33 analysis |
| Risk factors for severe pediatric food anaphylaxis in Italy | 2011 | PEDIATRIC ALLERGY AND IMMUNOLOGY | 22 | 8 | 813-819 | Calvani, M and Cardinale, F and Martelli, A and Muraro, A and Pucci, N and Savino, F and Zappalà, D and Panetta, V and Italian Soc Pediat Allergy Immunol | 10.1111/j.1399-3038.2011.01200.x     WE  - Science Citation Index Expanded (SCI-EXPANDED) | No IL-33 analysis |
| Allergenic food introduction and risk of childhood atopic diseases | 2017 | PLOS ONE | 12 | 11 | - | Elbert, NJ and Kiefte-de Jong, JC and Voortman, T and Nijsten, TEC and de Jong, NW and Jaddoe, VWV and de Jongste, JC and van Wijk, RG and Duijts, L and Pasmans, SGMA | 10.1371/journal.pone.0187999     WE  - Science Citation Index Expanded (SCI-EXPANDED) | No IL-33 analysis |
| Influence of age on IgE response in peanut-allergic children and adolescents from the Mediterranean area | 2015 | PEDIATRIC ALLERGY AND IMMUNOLOGY | 26 | 6 | 497-502 | Garcia-Blanca, A and Aranda, A and Blanca-Lopez, N and Perez, D and Gomez, F and Mayorga, C and Torres, MJ and Diaz-Perales, A and Perkins, JR and Villalba, M and Blanca, M and Canto, G | 10.1111/pai.12418     WE  - Science Citation Index Expanded (SCI-EXPANDED) | No IL-33 analysis |
| Resolution of childhood peanut allergy | 2000 | ANNALS OF ALLERGY ASTHMA & IMMUNOLOGY | 85 | 6 | 473-476 | Spergel, JM and Beausoleil, JL and Pawlowski, NA | 10.1016/S1081-1206(10)62574-4     WE  - Science Citation Index Expanded (SCI-EXPANDED) | No IL-33 analysis |
| Differentiating Peanut Allergy from Sensitization in Polish Children: A Real-Life Diagnostic Model | 2026 | LIFE-BASEL | 16 | 3 | - | Tworowska, J and Krogulska, A | 10.3390/life16030418     WE  - Science Citation Index Expanded (SCI-EXPANDED) | No IL-33 analysis |
| Desensitization and remission after peanut sublingual immunotherapy in 1-to 4-year-old peanut-allergic children: A randomized, placebo-controlled trial | 2024 | JOURNAL OF ALLERGY AND CLINICAL IMMUNOLOGY | 153 | 1 | - | Kim, EH and Bird, JA and Keet, CA and Virkud, YV and Herlihy, L and Ye, P and Smeekens, JM and Guo, R and Yue, XH and Penumarti, A and Qaqish, B and Li, QF and Kulis, MD and Burks, AW | 10.1016/j.jaci.2023.08.032 | No IL-33 analysis |
| A PRELIMINARY-STUDY OF INTERLEUKIN-4 DETECTION IN ATOPIC PEDIATRIC AND ADULT PATIENTS - EFFECT OF DIETARY MODIFICATION | 1993 | PEDIATRIC ASTHMA ALLERGY & IMMUNOLOGY | 7 | 1 | 27-35 | FRIERI, M and MARTINEZ, S and AGARWAL, K and TROTTA, P | 10.1089/pai.1993.7.27     WE  - Science Citation Index Expanded (SCI-EXPANDED) | No IL-33 analysis |
| Follow-up to Adolescence after Early Peanut Introduction for Allergy Prevention | 2024 | NEJM EVIDENCE | 3 | 6 | - | Du Toit, G and Huffaker, MF and Radulovic, S and Feeney, M and Fisher, HR and Byron, M and Dunaway, L and Calatroni, A and Johnson, M and Foong, RX and Marques-Mejias, A and Bartha, I and Basting, M and Brough, HA and Baloh, C and Laidlaw, TM and Bahnson, HT and Roberts, G and Plaut, M and Wheatley, LM and Lack, G and Immune Tolerance Network LEAP Trio Trial Team | 10.1056/EVIDoa2300311     WE  - Emerging Sources Citation Index (ESCI) | No IL-33 analysis |
| Defining the window of opportunity and target populations to prevent peanut allergy | 2023 | JOURNAL OF ALLERGY AND CLINICAL IMMUNOLOGY | 151 | 5 | 1329-1336 | Roberts, G and Bahnson, HT and Du Toit, G and O'Rourke, C and Sever, ML and Brittain, E and Plaut, M and Lack, G | 10.1016/j.jaci.2022.09.042 | No IL-33 analysis |
| Efficacy and Safety of Dupilumab in Children With Peanut Allergy: A Multicenter, Open-Label, Phase II Study | 2025 | ALLERGY | 80 | 1 | 227-237 | Sindher, SB and Nadeau, KC and Chinthrajah, RS and Leflein, JG and Bégin, P and Ohayon, JA and Ponda, P and Wambre, E and Liu, JZ and Khokhar, FA and Akinlade, B and Maloney, J and Orengo, JM and Hamilton, JD and Kamal, MA and Hooper, AT and Patel, N and Patel, K and Laws, E and Mannent, LP and Radin, AR | 10.1111/all.16404 | No IL-33 analysis |
| Dupilumab as an Adjunct to Oral Immunotherapy in Pediatric Patients With Peanut Allergy | 2025 | ALLERGY | 80 | 3 | 827-842 | Chinthrajah, RS and Sindher, SB and Nadeau, KC and Leflein, JG and Spergel, JM and Petroni, DH and Jones, SM and Casale, TB and Wang, J and Carr, WW and Shreffler, WG and Wood, RA and Wambre, E and Liu, JZ and Akinlade, B and Atanasio, A and Orengo, JM and Hamilton, JD and Kamal, MA and Hooper, AT and Patel, K and Laws, E and Mannent, LP and Adelman, DC and Ratnayake, A and Radin, AR | 10.1111/all.16420 | No IL-33 analysis |
| Effect of Epicutaneous Immunotherapy vs Placebo on Reaction to Peanut Protein Ingestion Among Children With Peanut Allergy The PEPITES Randomized Clinical Trial | 2019 | JAMA-JOURNAL OF THE AMERICAN MEDICAL ASSOCIATION | 321 | 10 | 946-955 | Fleischer, DM and Greenhawt, M and Sussman, G and Bégin, P and Nowak-Wegrzyn, A and Petroni, D and Beyer, K and Brown-Whitehorn, T and Hebert, J and Hourihane, JO and Campbell, DE and Leonard, S and Chinthrajah, RS and Pongracic, JA and Jones, SM and Lange, L and Chong, H and Green, TD and Wood, R and Cheema, A and Prescott, SL and Smith, P and Yang, W and Chan, ES and Byrne, A and Assa'ad, A and Bird, JA and Kim, EH and Schneider, L and Davis, CM and Lanser, BJ and Lambert, R and Shreffler, W | 10.1001/jama.2019.1113     WE  - Science Citation Index Expanded (SCI-EXPANDED)     WE  - Conference Proceedings Citation Index - Science (CPCI-S) | No IL-33 analysis |
| Efficacy, Safety, and Quality of Life in a Multicenter, Randomized, Placebo-Controlled Trial of Low-Dose Peanut Oral Immunotherapy in Children with Peanut Allergy | 2019 | JOURNAL OF ALLERGY AND CLINICAL IMMUNOLOGY-IN PRACTICE | 7 | 2 | 479-+ | Blumchen, K and Trendelenburg, V and Ahrens, F and Gruebl, A and Hamelmann, E and Hansen, G and Heinzmann, A and Nemat, K and Holzhauser, T and Roeder, M and Rosenfeld, L and Hartmann, O and Niggemann, B and Beyer, K | 10.1016/j.jaip.2018.10.048     WE  - Science Citation Index Expanded (SCI-EXPANDED) | No IL-33 analysis |
| Oral Immunotherapy for Peanut Allergy in Children 1 to Less Than 4 Years of Age | 2023 | NEJM EVIDENCE | 2 | 11 | - | Du Toit, G and Brown, KR and Vereda, A and Irani, AM and Tilles, S and Ratnayake, A and Jones, SM and Vickery, BP and POSEIDON Study Grp | 10.1056/EVIDoa2300145     WE  - Emerging Sources Citation Index (ESCI) | No IL-33 analysis |
| Safety of Epicutaneous Immunotherapy in Peanut-Allergic Children: REALISE Randomized Clinical Trial Results | 2022 | JOURNAL OF ALLERGY AND CLINICAL IMMUNOLOGY-IN PRACTICE | 10 | 7 | 1864-+ | Pongracic, JA and Gagnon, R and Sussman, G and Siri, D and Oriel, RC and Brown-Whitehorn, TF and Green, TD and Campbell, DE and Anvari, S and Berger, WE and Bird, JA and Chan, ES and Cheema, A and Chinthrajah, RS and Chong, HJ and Dowling, PJ and Fineman, SM and Fleischer, DM and Gonzalez-Reyes, E and Kim, EH and Lanser, BJ and MacGinnitie, A and Mehta, H and Petroni, D and Rupp, N and Schneider, LC and Scurlock, AM and Sher, LD and Shreffler, WG and Sindher, SB and Stillerman, A and Wood, R and Yang, WH and Bois, T and Sampson, HA and Bégin, P | 10.1016/j.jaip.2021.11.017 | No IL-33 analysis |
| First Real-World Effectiveness Analysis of Preschool Peanut Oral Immunotherapy | 2021 | JOURNAL OF ALLERGY AND CLINICAL IMMUNOLOGY-IN PRACTICE | 9 | 3 | 1349-+ | Soller, L and Abrams, EM and Carr, S and Kapur, S and Rex, GA and Leo, S and McHenry, M and Vander Leek, TK and Yeung, J and Cook, VE and Wong, T and Hildebrand, KJ and Mak, R and Gerstner, TV and Cameron, SB and Chan, ES | 10.1016/j.jaip.2020.10.045 | No IL-33 analysis |
| A pilot study of omalizumab to facilitate rapid oral desensitization in high-risk peanut-allergic patients | 2013 | JOURNAL OF ALLERGY AND CLINICAL IMMUNOLOGY | 132 | 6 | 1368-1374 | Schneider, LC and Rachid, R and LeBovidge, J and Blood, E and Mittal, M and Umetsu, DT | 10.1016/j.jaci.2013.09.046     WE  - Science Citation Index Expanded (SCI-EXPANDED) | No IL-33 analysis |
| A randomized controlled study of peanut oral immunotherapy: Clinical desensitization and modulation of the allergic response | 2011 | JOURNAL OF ALLERGY AND CLINICAL IMMUNOLOGY | 127 | 3 | 654-660 | Varshney, P and Jones, SM and Scurlock, AM and Perry, TT and Kemper, A and Steele, P and Hiegel, A and Kamilaris, J and Carlisle, S and Yue, XH and Kulis, M and Pons, L and Vickery, B and Burks, AW | 10.1016/j.jaci.2010.12.1111     WE  - Science Citation Index Expanded (SCI-EXPANDED) | No IL-33 analysis |
| Food-induced anaphylaxis in infants and children | 2018 | ANNALS OF ALLERGY ASTHMA & IMMUNOLOGY | 121 | 3 | 360-365 | Samady, W and Trainor, J and Smith, B and Gupta, R | 10.1016/j.anai.2018.05.025     WE  - Science Citation Index Expanded (SCI-EXPANDED) | No IL-33 analysis |
| Clinical efficacy and immune regulation with peanut oral immunotherapy | 2009 | JOURNAL OF ALLERGY AND CLINICAL IMMUNOLOGY | 124 | 2 | 292-300 | Jones, SM and Pons, L and Roberts, JL and Scurlock, AM and Perry, TT and Kulis, M and Shreffler, WG and Steele, P and Henry, KA and Adair, M and Francis, JM and Durham, S and Vickery, BP and Zhong, XP and Burks, AW | 10.1016/j.jaci.2009.05.022     WE  - Science Citation Index Expanded (SCI-EXPANDED) | No IL-33 analysis |
| Risk Factors for Anaphylaxis in Children Allergic to Peanuts | 2023 | MEDICINA-LITHUANIA | 59 | 6 | - | Petek, T and Lajhar, M and Krasovec, B and Homsak, M and Kavalar, M and Korosec, P and Koren, B and Tomazin, M and Hojnik, T and Berce, V | 10.3390/medicina59061037     WE  - Science Citation Index Expanded (SCI-EXPANDED) | No IL-33 analysis |
| Is the prevalence of peanut allergy increasing? A 5-year follow-up study in children in Montreal | 2009 | JOURNAL OF ALLERGY AND CLINICAL IMMUNOLOGY | 123 | 4 | 783-788 | Ben-Shoshan, M and Kagan, RS and Alizadehfar, R and Joseph, L and Turnbull, E and St Pierre, Y and Clarke, AE | 10.1016/j.jaci.2009.02.004     WE  - Science Citation Index Expanded (SCI-EXPANDED) | No IL-33 analysis |
| Peanut Allergy in Spanish Children: Comparative Profile of Peanut Allergy versus Tolerance | 2019 | INTERNATIONAL ARCHIVES OF ALLERGY AND IMMUNOLOGY | 178 | 4 | 370-376 | Ibarra, GZ and Aparicio, VF and Herrero, SI and Blanca, M and Remon, LZ | 10.1159/000495579     WE  - Science Citation Index Expanded (SCI-EXPANDED) | No IL-33 analysis |
| Food-induced anaphylaxis in early childhood and factors associated with its severity | 2021 | ALLERGY AND ASTHMA PROCEEDINGS | 42 | 5 | E135-E144 | Aydogan, M and Topal, E and Yakici, N and Acar, HC and Demirkale, ZH and Arga, M and Uysal, P and Aydemir, S and Simsek, IE and Tamay, Z and Cekic, S and Cavkaytar, O and Kaplan, F and Kiykim, A and Cogurlu, MT and Süleyman, A and Yücel, E and Akkelle, E and Hancioglu, G and Yasar, A and Tuncel, T and Nacaroglu, HT and Aydogmus, C and Güler, N and Cokugras, H and Sapan, N and Yüksel, H and Sancak, R and Erdogan, MS and Ozdemir, O and Ozdemir, C and Orhan, F | 10.2500/aap.2021.42.210051     WE  - Science Citation Index Expanded (SCI-EXPANDED) | No IL-33 analysis |
| Correlation of allergen-specific IgG subclass antibodies and T lymphocyte cytokine responses in children with multiple food allergies | 2010 | PEDIATRIC ALLERGY AND IMMUNOLOGY | 21 | 6 | 935-944 | Scott-Taylor, TH and Hourihane, JO and Strobel, S | 10.1111/j.1399-3038.2010.01025.x     WE  - Science Citation Index Expanded (SCI-EXPANDED) | No IL-33 analysis |
| Patterns of food allergen-specific cytokine production by T lymphocytes of children with multiple allergies | 2005 | CLINICAL AND EXPERIMENTAL ALLERGY | 35 | 11 | 1473-1480 | Scott-Taylor, TH and Hourihane, JB and Harper, J and Strobel, S | 10.1111/j.1365-2222.2005.02355.x     WE  - Science Citation Index Expanded (SCI-EXPANDED) | No IL-33 analysis |
| Association of T helper type 2 cytokines with sensitization to food in pediatric atopic dermatitis patients | 2019 | EGYPTIAN JOURNAL OF PEDIATRIC ALLERGY AND IMMUNOLOGY | 17 | 2 | 69-76     WE  - Emerging Sources Citation Index (ESCI) | Paulauskaite, I and Eidukaite, A and Rudzeviciene, O and Orentaite, R |  | Wrong study population |
| Phase 3 Trial of Epicutaneous Immunotherapy in Toddlers with Peanut Allergy | 2023 | NEW ENGLAND JOURNAL OF MEDICINE | 388 | 19 | 1755-1766 | Greenhawt, M and Sindher, SB and Wang, J and O'Sullivan, | 10.1056/NEJMoa2212895     WE  - Science Citation Index Expanded (SCI-EXPANDED) | No IL-33 analysis IL33 |
| IgG4 inhibits peanut-induced basophil and mast cell activation in peanut-tolerant children sensitized to peanut major allergens | 2015 | JOURNAL OF ALLERGY AND CLINICAL IMMUNOLOGY | 135 | 5 | 1249-1256 | Santos, AF and James, LK and Bahnson, HT and Shamji, MH and Couto-Francisco, NC and Islam, S and Houghton, S and Clark, AT and Stephens, A and Turcanu, V and Durham, SR and Gould, HJ and Lack, G | 10.1016/j.jaci.2015.01.012     WE  - Science Citation Index Expanded (SCI-EXPANDED) | No IL-33 analysis |
| IL-33/s-ST2 ratio, systemic symptoms, and basophil activation in Pru p 3-sensitized allergic patients | 2018 | Inflammation Research | 67 | 8 | 671-679 | Uasuf, C.G. and Di Sano, C. and Gangemi, S. and Albeggiani, G. and Cigna, D. and Dino, P. and Brusca, I. and Gjomarkaj, M. and Pace, E. | 10.1007/s00011-018-1157-4 | wrong population |
| Mechanical Skin Injury Promotes Food Anaphylaxis by Driving Intestinal Mast Cell Expansion | 2019 | Immunity | 50 | 5 | 1262-1275.e4 | Leyva-Castillo, J.-M. and Galand, C. and Kam, C. and Burton, O. and Gurish, M. and Musser, M.A. and Goldsmith, J.D. and Hait, E. and Nurko, S. and Brombacher, F. and Dong, C. and Finkelman, F.D. and Lee, R.T. and Ziegler, S. and Chiu, I. and Austen, K.F. and Geha, R.S. | 10.1016/j.immuni.2019.03.023 | : wrong population |
| Microneedles Coated with Cow’s Milk Proteins: Immunogenicity, Stability, and Safety Assessment | 2025 | Molecular Pharmaceutics | 22 | 6 | 2858-2867 | Venkatesa Prabhu, G.K. and Shakya, A.K. and Gill, H.S. | 10.1021/acs.molpharmaceut.4c01136 | No IL-33 analysis |
| TSLP, IL-31, IL-33 and sST2 are new biomarkers in endophenotypic profiling of adult and childhood atopic dermatitis | 2016 | Journal of the European Academy of Dermatology and Venereology | 30 | 11 | 1930-1938 | Nygaard, U. and Hvid, M. and Johansen, C. and Buchner, M. and Fölster-Holst, R. and Deleuran, M. and Vestergaard, C. | 10.1111/jdv.13679 | wrong population |
| Increased miR-21-3p and miR-487b-3p serum levels during anaphylactic reaction in food allergic children | 2021 | Pediatric Allergy and Immunology | 32 | 6 | 1296-1306 | Nuñez-Borque, E. and Fernandez-Bravo, S. and Rodriguez Del Rio, P. and Alwashali, E.M. and Lopez-Dominguez, D. and Gutierrez-Blazquez, M.D. and Laguna, J.J. and Tome-Amat, J. and Gallego-Delgado, J. and Gomez-Lopez, A. and Betancor, D. and Cuesta-Herranz, J. and Ibañez-Sandin, M.D. and Benito-Martin, A. and Esteban, V. | 10.1111/pai.13518 | No IL-33 analysis |
| The influence of the microbiome on allergic sensitization to food | 2017 | Journal of Immunology | 198 | 2 | 581-589 | Plunkett, C.H. and Nagler, C.R. | 10.4049/jimmunol.1601266 | No IL-33 analysis |
| Biomarkers of T2 inflammation in the diagnosis and monitoring of eosinophilic esophagitis in children | 2025 | Russian Journal of Allergy | 22 | 2 | 122-134 | Vyazankina, S.S. and Makarova, S.G. and Lokhmatov, M.M. and Murashkin, N.N. and Budkina, T.N. and Semikina, E.L. and Snovskaya, M.A. and Kurbatova, O.V. and Zhuzhula, A.A. and Fisenko, A.P. and Ereshko, O.A. and Ivanova, V.M. | 10.36691/RJA16994 | Non- English paper |
| Mechanisms for Alternaria alternata Function in the Skin During Induction of Peanut Allergy in Neonatal Mice With Skin Barrier Mutations | 2021 | Frontiers in Allergy | 2 | 677019 | Article 677019 | Buelow, L.M. and Hoji, A. and Tat, K. and Schroeder-Carter, L.M. and Carroll, D.J. and Cook-Mills, J.M. | 10.3389/falgy.2021.677019 | Article banned due to academic integrity |
| Efficacy and safety of oral immunotherapy in children aged 1-3 years with peanut allergy (the Immune Tolerance Network IMPACT trial): a randomised placebo-controlled study | 2022 | The Lancet | 399 | 10322 | 359–371 | Jones SM, Kim EH, Nadeau KC, Nowak-Wegrzyn A, Wood RA, Sampson HA, Scurlock AM, Chinthrajah S, Wa... and Immune Tolerance Network. | 10.1016/S0140-6736(21)02390-4 | No IL-33 analysis |
| Constitutively high levels of endogenous soluble ST2 inhibit food allergic responses in mice | 2025 | Proceedings of the National Academy of Sciences of the United States of America | 122 | 47 | e2506485123 | Kojima M, Izawa K, Ando T, Maeda K, Kaitani A, Nakano N, Yamamoto R, Miyazaki S, Shinagawa M, Sas... | 10.1073/pnas.2506485123 | No IL-33 analysis |
| Phase 2a randomized, placebo-controlled study of anti-IL-33 in peanut allergy | 2020 | JCI Insight | 5 | 22 | e131347 | Chinthrajah S, Cao S, Liu C, Lyu SC, Sindher SB, Long A, Sampath V, Petroni D, Londei M, Nadeau KC. | 10.1172/jci.insight.131347 | wrong population |
| Temporal Regulation by Innate Type 2 Cytokines in Food Allergies | 2017 | Current Allergy and Asthma Reports | 17 | 1 | 2 | Graham MT, Andorf S, Spergel JM, Chatila TA, Nadeau KC. | 10.1007/s11882-016-0656-z | Wrong publication type |
| Mass cytometry reveals cellular fingerprint associated with IgE+ peanut tolerance and allergy in early life | 2020 | Nature Communications | 11 | 1 | 1095 | Neeland MR, Andorf S, Manohar M, Dunham D, Lyu SC, Dang TD, Peters RL, Perrett KP, Tang MLK, Saff... | 10.1038/s41467-020-14919-4 | No IL-33 analysis |
| Uric acid pathway activation during respiratory virus infection promotes Th2 immune response via innate cytokine production and ILC2 accumulation | 2020 | Mucosal Immunology | 13 | 3 | 691–701 | Fonseca W, Malinczak CA, Schuler CF, Best SKK, Rasky AJ, Morris SB, Cui TX, Popova AP, Lukacs NW. | 10.1038/s41385-020-0264-z | No IL-33 analysis |
| Patch testing of food allergens promotes Th17 and Th2 responses with increased IL-33: a pilot study | 2016 | Experimental Dermatology | 25 | 6 | 469-471 | Ungar B, Correa da Rosa J, Shemer A, Czarnowicki T, Estrada YD, Fuentes-Duculan J, Xu H, Zheng X,... | 10.1111/exd.13148 | wrong population type |

**Abbreviations:** AD, atopic dermatitis; EoE, eosinophilic esophagitis; EGE, eosinophilic gastroenteritis; EGID, eosinophilic gastrointestinal disorder; FA, food allergy; HDM, house dust mite; IgE, immunoglobulin E; IL-33, interleukin-33; OIT, oral immunotherapy; sST2, soluble suppression of tumorigenicity 2; ST2, suppression of tumorigenicity 2; TSLP, thymic stromal lymphopoietin. Studies were excluded after full-text assessment according to the prespecified eligibility criteria. A single primary reason for exclusion is reported for each study.
